# Conferring of Drought and Heat Stress Tolerance in Wheat (*Triticum aestivum* L.) Genotypes and Their Response to Selenium Nanoparticles Application

**DOI:** 10.3390/nano13060998

**Published:** 2023-03-09

**Authors:** Ahmad A. Omar, Yasmin M. Heikal, Ehab M. Zayed, Sahar A. M. Shamseldin, Yossry E. Salama, Khaled E. Amer, Mostafa M. Basuoni, Sawsan Abd Ellatif, Azza H. Mohamed

**Affiliations:** 1 Biochemistry Department, Faculty of Agriculture, Zagazig University, Zagazig 44519, Egypt; 2 Citrus Research and Education Center, University of Florida, IFAS, Lake Alfred, FL 33850, USA; 3 Botany Department, Faculty of Science, Mansoura University, Mansoura 35516, Egypt; 4 Cell Study Research Department, Field Crops Research Institute, Agricultural Research Center, Giza 12619, Egypt; 5 Botany Department, Women’s College for Arts, Science and Education, Ain Shams University, Cairo 11566, Egypt; 6 Crop Science Department, Faculty of Agriculture, Damanhour University, Damanhour 22516, Egypt; 7 Botany and Microbiology Department, Faculty of Science (Boys), Al-Azhar University, Cairo 11884, Egypt; 8 Bioprocess Development Department, Genetic Engineering and Biotechnology Research Institute (GEBRI), City for Scientific Research and Technology Applications, New Borg El-Arab 21934, Egypt; 9 Agricultural Chemistry Department, Faculty of Agriculture, Mansoura University, Mansoura 33516, Egypt

**Keywords:** selenium nanoparticles, wheat, drought, heat, antioxidant enzymes, gene expression

## Abstract

In this study, the role of selenium nanoparticles (SeNPs, 10 mg·L^−1^) has been investigated in modulating the negative effects of drought and heat stresses on eight bread wheat (*Triticum aestivum* L.) genotype seedlings. Those genotypes included Giza-168, Giza-171, Misr-1, Misr-3, Shandweel-1, Sids-1, Sids-12, and Sids-14. The study included six treatments as follows: regular irrigation with 100% Field Capacity (FC) at a temperature of 23 ± 3 °C (T1), drought stress with 60% FC (T2), heat stress of 38 °C for 5 h·day^−1^ (T3), foliar spray of 10 mg·L^−1^ of SeNPs only (T4), a combination of drought stress with foliar spray of 10 mg·L^−1^ of SeNPs (T5), and heat stress with foliar spray of 10 mg·L^−1^ of SeNPs (T6). The experiment continued for 31 days. Foliar application of SeNPs improved the plant growth, morpho-physiological and biochemical responses, and expression of stress-responsive genes in wheat (*T. aestivum* L.) seedlings. Overall, morpho-physiological traits such as plant height (PH), shoot fresh weight (SFW), shoot dry weight (SDW), root fresh weight (RFW), and root dry weight (RDW) of wheat genotypes grown under different conditions ranged from 25.37–51.51 cm, 3.29–5.15 g, 0.50–1.97 g, 0.72–4.21 g, and 0.11–1.23 g, respectively. From the morpho-physiological perspective, drought stress had a greater detrimental impact on wheat plants than heat stress, whereas heat stress significantly impacted the expression of stress-responsive genes. Stress responses to drought and heat varied between wheat genotypes, suggesting that different genotypes are more resilient to stress. Exogenous spraying of 10 mg·L^−1^ of SeNPs improved the photosynthetic pigments, photosynthetic rate, gas exchange, and transpiration rate of wheat plants and enhanced drought and heat tolerance by increasing the activity of antioxidant enzymes including catalase (CAT), ascorbate peroxidase (APX), and superoxide dismutase (SOD) and the expression level of stress-responsive genes. Our results showed that spraying wheat seedlings with 10 mg·L^−1^ of SeNPs enhanced SOD activity for all genotypes as compared to the control, with the Sids-12 genotype having the highest value (196.43 U·mg^−1^ FW·min^−1^) and the Giza-168 genotype having the lowest (152.30 U·mg^−1^ FW·min^−1^). The expression of *PIP1*, *LEA-1*, *HSP70*, and *HSP90* stress-responsive genes was more significant in tolerant genotypes (Giza-171 and Giza-168) than in sensitive ones (Misr-1 and Misr-3) in response to drought and heat stresses. Under stress conditions, the shoot and root fresh weights, photosynthetic pigment content, stomatal conductance (SC), and transpiration rate (TR) were positively correlated with plant height (PH), while root and shoot dry weights, malondialdehyde (MDA), proline, hydrogen peroxide (H_2_O_2_), and APX were negatively correlated. Multivariate analysis and biplot results revealed that genotypes Giza-168, Giza-171, Sids-12, and Sids-14 performed well in both stress situations and were classified as stress-tolerant genotypes. These best genotypes may be employed in future breeding projects as tools to face climate change. This study concluded that various physio-biochemicals and gene expression attributes under drought and heat stress could be modulated by foliar application of SeNPs in wheat genotypes, potentially alleviating the adverse effects of drought and heat stress.

## 1. Introduction

Globally, wheat (*Triticum aestivum* L.) is a staple crop. The global wheat industry faces numerous challenges, including the convergence of genetic resources in behavior and productivity, biotic and abiotic stress, soil poverty, and an increase in global consumption because of an increase in the world population, global supply chains, and a monopoly on fertilizers for better agricultural practices. According to [1], wheat demand will increase by 60% by 2050. Abiotic stresses such as drought, salinity, and heat limit wheat productivity [2]. According to previous studies, wheat yield is negatively impacted by periodic drought stress during the growth and development seasons under a wide climate zone, especially in arid and semi-arid climates [3]. Consequently, the selection and breeding of more drought-tolerant wheat genotypes need to be undertaken to expand wheat production in arid and semi-arid areas [4]. Water poverty, climatic changes, the convergence of genetic content, and increased demand for wheat grain are driving forces to enhance wheat productivity [5,6]. Egypt is one of the world’s largest wheat consumers and imports about 22 million tons (USDA report, https://www.fas.usda.gov/data/egypt-grain-and-feed-update-5, accessed on 20 January 2023).

The frequency of droughts and heat during the various phases of crop growth has increased in recent decades. When drought and elevated temperatures are present, crop yield could be reduced by up to 50%; therefore, plants activate different morphological, physiological, and biochemical pathways for stress management [7,8]. A plant’s breeding and molecular strategies for dealing with low water availability and high temperatures are avoidance and tolerance, with a particular focus on changing omics technology [9]. The plants manage drought and heat stress through the balance between transpiration, respiration, photosynthesis, and the ability to adapt to and tolerate the stress condition [8,10]. Drought and heat constrain wheat yields by reducing grain formation and development [11]. Heat stress also impacts wheat growth, photosynthesis, and pollen fertility [11].

Physiological and biochemical changes occur in wheat plants due to drought stress, including hormonal imbalance, ethylene production, and reduced photosynthesis, because drought affects cell and tissue structure and important processes at all stages of growth [12]. As a result, oxidative damage and membrane lipid peroxidation occur, which leads to cell death and plant tissue damage in addition to the general wilting of the plant from this point on [12]. On the other hand, stress affects crops differently depending on their intensity, duration, and stages, where the genetic makeup of crops plays an important role in addition to the environment that bears the type of stress and the plant’s adaptation to it [13]. In drought conditions, wheat plants respond physiologically to water deficit by synthesizing and accumulating compatible osmolytes and osmoprotectant compounds that lower the water potential within their cells and enhance water extraction from the root system, so the fresh weight of the root is a very important trait to the potential for tolerance [12]. Recent research innovations are trending around smart nano-formulations that improve crop protection and plant nutrition [14]. It has been shown that nanoparticles (NPs) can increase plant tolerance to abiotic stress conditions much more quickly by improving morphological traits through physiological and biochemical processes and increasing gene expression [15,16]. The use of nanotechnology will enable sustainable agricultural development, enabling a green revolution in agricultural production through the use of green and safe nanomaterials. Moreover, the synthesized green nanomaterials will be cost-effective, driving the agricultural economy [17]. The use of nanotechnology and engineered nanomaterials in the food and agriculture (nano agrifood) sectors is intended to provide several potential benefits and opportunities that align with the principles of responsible innovation [18]. Nanoparticles alleviate and improve the negative effects of environmental stresses on wheat plants while improving their quality by increasing the vital processes in the plant [19]. Among various nanoparticles (NPs), selenium nanoparticles (SeNPs) at low concentrations were found to have prominent bioactivity and biosafety properties and were known to be nontoxic and biocompatible compared to their counterparts, selenate (SeO_4_^−2^) and selenite (SeO_3_^−2^). Size reduction will also boost the activity of nanoparticles and make them more efficient [20].

According to the best of our knowledge, there are no reports available about the drought or heat tolerance status at the seedling stage of the investigated wheat genotypes. Therefore, the current study aimed to screen the selected wheat genotypes for drought and/or heat tolerance and to investigate the impacts of chemically synthesized SeNPs on the physiology, biochemistry, and expression level of stress-responsive genes induced by drought and heat stress in the eight wheat genotypes chosen for this investigation. This could help identify the characteristics that influence the drought and heat tolerance of wheat plants for use in breeding programs in the future.

## 2. Materials and Methods

### 2.1. Selenium Nanoparticles and Bread Wheat Seeds Pedigree

The SeNPs used in this study were obtained from Sigma-Aldrich (Taufkirchen, Germany) in physical dispersion form, with average particle sizes ranging from 10 to 100 nm and a concentration of 0.15% of selenium in water. Eight bread wheat (*T. aestivum* L.) genotypes: Giza-168, Giza-171, Misr-1, Misr-3, Shandweel-1, Sids-1, Sids-12, and Sids-14 were obtained from the Wheat Research Department, Field Crops Research Institute, Agricultural Research Center (ARC), Ministry of Agriculture, Egypt. These genotypes are the most widely used by Egyptian farmers and in the local Egyptian markets. The names of these genotypes and pedigrees are listed in Table S1 from Supplementary Materials.

### 2.2. Characterization of SeNPs

#### 2.2.1. Transmission Electron Microscopy

The form and size of selenium nanoparticles were investigated using transmission electron microscopy (TEM) (JEOL JEM-2100 equipment, Akishima, Tokyo, Japan). An aqueous suspension containing the SeNPs was dispersed ultrasonically, and the sample was prepared by placing a drop of the solution on a copper grid coated with carbon and drying it under an IR lamp. Micrographs were obtained using a TEM operated at an accelerating voltage of 80 kV. The related size distribution pattern of SeNPs was plotted by manually counting 200 individual particles from different TEM images.

#### 2.2.2. Fourier Transform Infrared (FTIR) Analysis

Fourier transform infrared spectroscopy analysis was done to characterize the functional groups on the surface of SeNPs. A powder sample of SeNPs was used for FTIR spectroscopy measurements. Powder and potassium bromide (KBr) were mixed in a 1:100 ratio, and a Shimadzu FTIR (Shimadzu 8400S, Chiyoda-Ku, Tokyo, Japan) apparatus was used to record the spectra. The FTIR spectrum was obtained with wave numbers between 500 cm^−1^–4000 cm^−1^.

### 2.3. Experimental Design and Culture Condition

A pot experiment was conducted at the greenhouse of Scientific Research City and Technological Applications (SRTA-City), New Borg El Arab City, Egypt, to examine the impact of SeNPs on the morpho-physiological, biochemical, and molecular responses of eight bread wheat genotypes growing under drought and heat stress conditions. In plastic pots (10 cm diameter × 12 cm depth), filled with soil, clay, and compost (4:1), and adding 10 g of N, K, and P (10-5-5) nutrients. After wheat seed sterilization, we sowed 20 homogenous, healthy seeds in each pot and submerged the pots with ¼ strength Hoagland nutrient-supplemented irrigation water [21] every other day for 1 week (7 days after sown, DAS).

The greenhouse photoperiod of 13/11 h (light/dark), air relative humidity of 65 ± 5%, and (day/night) temperature were maintained at 23/17 ± 3 °C throughout the pot experiments other than the heat stress condition. The treatments were as follows: T1, control (regular irrigation, 100% Field Capacity (FC)) at (day/night) temperature of 23/17 ± 3 °C; T2, drought stress (60% FC); T3, heat stress (38 °C) with regular irrigation (100% FC); T4, 10 mg·L^−1^ of SeNPs with regular irrigation (100% FC) and temperature of 23/17 ± 3 °C; T5, drought stress (60% FC) + 10 mg·L^−1^ of SeNPs; and T6, heat stress (38 °C) + 10 mg·L^−1^ of SeNPs. All those treatment conditions were examined in pot experiments with three replicates for each treatment, which continued until the end of the experiment. After 1 week (7 DAS), the germinated wheat seedlings were reduced to 8 wheat seedlings per pot (thinning) to have homogeneity among all pots and continued germination for another 2 weeks (21 DAS). During these 2 weeks, the drought stress treatments (T2 and T5) were with moderate drought (60% FC) by irrigation every 3 days, and the heat stress treatments (T3 and T6) were at 38 °C (5 h·day^−1^). Finally, at 21 DAS, all pots were moved to the greenhouse standard conditions (regular irrigation (100% FC) and 23/17 ± 3 °C), and an exogenous application of 10 mg·L^−1^ of SeNPs (20 mL·pot^−1^) was applied to T4, T5, and T6 at 3-day intervals for 10 days. Distilled water was used for the control treatment (T1). At the end of the experiment (31 DAS), samples were collected for further analysis. SeNPs concentration (10 mg·L^−1^) was selected in this study according to previous studies that recommended using 30 mg·L^−1^ or less of SeNPs. The SeNPs at a concentration of 30 mg·L^−1^ or less were found to be the most suitable concentrations that enhanced the morphological, physiological, biochemical, and antioxidant parameters under different biotic and abiotic stresses [22]. Additionally, Zhang et al. [23] used *Spirulina platensis* polysaccharides (SPs) as stabilizers to prepare SPs-SeNPs and reported that the median toxic concentration (TC_50_) of SPs-SeNPs was approximately 22,000 μg·L^−1^. Three replicates for each treatment were established (Table 1).

### 2.4. Measurement of Morphological Traits and Growth Parameters

At the end of the experiment (31 DAS), plant samples from three separated pots in each treatment were harvested. To estimate growth and physiological parameters, three wheat plants from each treatment were taken and washed with distilled water to remove soil and adhering particles. A measuring scale immediately assessed plant height (PH) (cm) after harvest. Calculations were made for shoot fresh weight (SFW) and root fresh weight (RFW). To clean the root for mass analysis, the root system was held at the plant base and gently shaken free of bulk soil. The root was then transferred to a clean sheet of paper, and the soil that was not rigidly attached to the roots was gently brushed off with a soft brush. The soil that remained attached to roots after this treatment was considered rhizosheath soil. After that, the roots were washed over a 0.5-mm mesh, blotted, and weighed to obtain the fresh root mass. Additionally, after oven-drying at 65 °C for 48 h, the shoot dry weight (SDW) and root dry weight (RDW) were determined. The number of leaves per plant and leaf area (cm^2^) per plant were measured at the final harvesting date.

### 2.5. Determination of Chlorophyll Content, Relative Water Relations and Gas Exchange Parameters

According to Arnon, chlorophyll content (CHLO) in the uppermost fully expanded leaves was determined spectrophotometrically [24]. A leaf sample (200 mg) was incubated for 10 min in aqueous acetone (80%) and centrifuged for 10 min at 12,000× *g*. The absorbance of the clear solvent was recorded at 663 and 645 nm using a UV/VIS spectrophotometer (Jenway 6305 Benchtop UV/Visible Spectrophotometer, Cole-Parmer UK, Cambridgeshire, UK). The net photosynthetic rate (PN), stomatal conductance (SC), and transpiration rate (TR) were measured using an infrared gas analyzer system (TPS-2, Portable Photosynthesis System, Amesbury, MA, USA). These parameters were measured in the middle region of the uppermost fully expanded leaves of treated and non-treated wheat plants on a bright sunny day between 9:30 and 11:30 a.m.

Electrolyte leakage (EL) was measured by boiling fresh leaf discs in 10 mL of deionized water, and electrical conductivity (EC1) was measured. After that, tubes were heated at 55 °C for 30 min, and electrical conductivity (EC2) was measured again. Again, after boiling the tissue for 10 min at 100 °C, electrical conductivity (EC3) was recorded [25]. The calculation was done using the following formula:

(1) $$\mathrm{Electrolyte}\;\mathrm{Leakage}\;\left(\%\right)=\frac{\mathrm{EC}2-\mathrm{EC}1}{\mathrm{EC}3}\times 100$$

The method of Sun et al. [26] was used to determine the relative water content (RWC), and it was calculated using the following formula:

(2) $$RWC\;\left(\%\right)=\frac{FW-DW}{TW-DW}\times 100$$

On fully expanded leaves of physiological activity-controlled wheat plants and wheat plants under stress, leaf water potential (Ψ) was measured. Then, based on the formula described by Campos et al. [27], the water use efficiency (WUE) was calculated.

### 2.6. Estimation of Stress Induced Biomarkers: Hydrogen Peroxide (H_2_O_2_) Levels, Malondialdehyde (MDA) and Proline

The H_2_O_2_ levels were measured according to the previously described method [28]. Briefly, leaf samples were extracted with trichloroacetic acid (TCA) and centrifuged at 12,000× *g* for 15 min. Then, in a reaction mixture of 0.5 mL enzyme supernatants, 0.5 mL phosphate buffer (pH 7.0), and 1 mM potassium iodide, the absorbance of the mixture was measured at 390 nm against H_2_O_2_ as a standard.

A method described by Heath and Packer [29] was used to measure MDA in *T. aestivum* fresh leaves. Briefly, 0.5 g of leaf samples were homogenized in 10 mL of ethanol and then centrifuged for 10 min at 10,000× *g*. An amount of 1 milliliter of the extract was added to 2 milliliters of 0.65% thiobarbituric acid (TBA) in 20% TCA. After boiling for 30 min, the mixture was rapidly cooled. Following centrifugation of the samples at 10,000× *g* for 5 min. The MDA content was determined by comparing non-specific absorption at 600 and 532 nm.

Proline was determined by extracting 500 mg of dried powdered leaf tissue in 3% sulphosalicylic acid. Following centrifugation of the extract for 20 min at 3000× *g*, 2 mL of the supernatant was added to 2 mL glacial acetic acid and 2 mL of the ninhydrin reagent, and the reaction was incubated at 100 °C for 1 h. Afterward, tubes were kept in an ice bath, and toluene was used to separate the proline. An optical density of 520 nm was then determined [30].

### 2.7. Assay of Antioxidant Enzymes

Using a prechilled pestle and mortar, we homogenized 1 g of fresh leaf tissue in a chilled 50 mM phosphate buffer (pH 7.0) with 1% polyvinyl pyrrolidone and 1 mM EDTA to extract the antioxidant enzymes. To obtain enzyme sources, the homogenate was centrifuged at 15,000× *g* for 20 min at 4 °C. The activity of superoxide dismutase (SOD) was determined by the reduction of nitroblue tetrazolium (NBT). The SOD was measured at 560 nm in an assay mix containing sodium phosphate buffer (50 mM, pH 7.5), 100 µL EDTA, L-methionine, 75 µM NBT, riboflavin, and 100 µL enzyme extract [31]. One unit of enzyme activity was defined as the amount of enzyme required for 50% inhibition of the rate of nitroblue tetrazolium reduction measured at 560 nm.

To determine ascorbate peroxidase (APX) activity, we monitored the absorption change in the rate of ascorbate oxidation at 290 nm for 3 min in a reaction mixture containing potassium phosphate buffer (pH 7.0), 0.5 mM ascorbic acid, hydrogen peroxide, and enzyme extract in a 1 mL reaction volume according to Nakano and Asada [32]. To determine catalase (CAT) activity, the method of Cakmak and Marschner [33] was followed, and the calculations were performed by measuring the decrease in absorbance at 240 nm to determine the disappearance of H_2_O_2_. The reaction mixture contained 25 mM phosphate buffer (pH 7.0), 10 mM H_2_O_2_, and 0.1 mL enzyme extract.

### 2.8. Quantitative Detection of Stress-Responsive Genes in Wheat Genotypes

Total RNA was isolated from a 0.5 g sample of wheat plants of all treatments by using the Plant RNA Kit (Sigma-Aldrich) according to the manufacturer’s instructions. The purified RNA was quantitated spectrophotometrically and analyzed on a 1.5% agarose gel. For each sample, 10 μg total RNA was treated with DNAse RNAse-free (Fermentas), 5 μg of which was reverse transcribed in a reaction mixture consisting of oligo dT primer (10 pmL·μL^−1^), 2.5 μL 5× buffer, 2.5 μL MgCl_2_, 2.5 μL 2.5 mM dNTPs, 4 μL from oligo (dT), 0.2 μL (5 Unit·μL^−1^) reverse transcriptase (Promega, Walldorf, Germany), and 2.5 μL RNA. The reverse transcription PCR (RT-PCR) amplification reaction was conducted in a thermal cycler PCR, programmed at 42 °C for 1 h and 72 °C for 20 min.

Quantitative Real-time PCR (QPCR) was conducted on 1 μL diluted cDNA in triplicate using real-time analysis using the Rotor-Gene™ 6000 system (LTF Labortechnik, Wasserburg, Germany). The primer sequences used in QPCR are listed in Table S2. Primers of four metal-tolerant, phytochelating, and Zn transporter genes and a housekeeping gene (reference gene) were used for gene expression analysis using a SYBR^®^ Green-based method. A total reaction volume of 20 µL was used. The reaction’s mixture consisted of 2 µL of cDNA template, 10 µL of SYBR Green Master Mix, 2 µL of each reverse and forward primer, and ddH_2_O for a total volume of 20 µL. PCR assays were performed using the following conditions: 95 °C for 15 min, followed by 40 cycles of 95 °C for 30 s and 60 °C for 30 s. The CT of each sample was used to calculate ΔCT values (target gene CT subtracted from β-Actin gene CT). The relative gene expression was determined using the 2^−ΔΔCt^ method [34].

### 2.9. Statistical Analysis

For morpho-physiological and molecular parameters among the genotypes, all data obtained were subjected to two-factor (treatment × genotypes) analysis of variance (ANOVA) using the general linear model, and the mean differences were compared using Tukey’s HSD test at *p* ≤ 0.05 using SPSS 16.0 (IBM, Armonk, NY, USA). Data were collected in a randomized complete block design with three replications. Histograms and violin plots were plotted using GraphPad Prism 9 (GraphPad Software, Inc., San Diego, CA, USA). Normality was assessed using Shapiro–Wilk normality testing at the 0.05 level. Correlation coefficient matrices adapted to compute normalized mean values for generating scatter plots and heatmaps of the morpho-physiological parameters among studied genotypes. Principal component analysis (PCA-biplot) is a multivariate approach that examines the distribution of eight wheat genotypes based on data matrices comprising many associated quantitative dependent variables. Additionally, PCA-biplots and the score plots PC1 and PC2 were conducted of all data under different conditions. The PCA-biplot analysis was performed using JMP^®^, Version 16 (SAS Institute Inc., Cary, NC, USA, 2020–2021).

## 3. Results

### 3.1. Characterization of SeNPs

The morphology of the SeNPs was observed using TEM, as shown in Figure S1A. The TEM micrograph clearly illustrated individual SeNPs with a small amount of aggregation. Most of the particles were spherical, and the irregular SeNPs had an average size of 5–70 ± 0.32 nm.

The FTIR analysis of SeNPs is provided in Figure S1B. A broad vibration peak reflected an O–H stretch of alcohols and phenols at 3335 cm^−1^. A small band at 2345 cm^−1^ is associated with nitro compounds (N-O asymmetric stretch) present in the mixture, and a strong band at 1652 cm^−1^ with the C=O stretching vibrations. The band at 1571 cm^−1^ was due to N–O asymmetric stretch nitro compounds. Two sharp peaks at 1126 and 991 cm^−1^ were associated with the bending vibrations of the CH_2_ groups. The 933 cm^−1^ represented the stretching of C-N bonds in amines. A band at 916 cm^−1^ resulted from carboxylic acid’s O–H bend. As a result of C–X stretching in alkyl halides, there were bands at 814 and 694 cm^−1^. According to these results, various functional groups related to surface proteins in SeNPs may be responsible for their reduction and stabilization.

### 3.2. Morphological Responses and Growth Parameters

**Figure 1:**
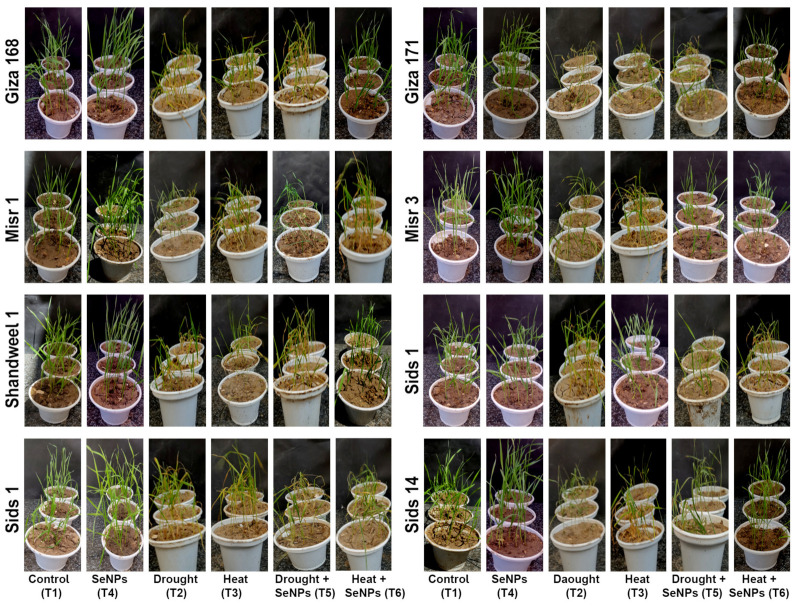
Morphological description of seedlings of eight wheat genotypes seedlings at 31 DAS grown under different growth conditions. Treatments: T1, control (regular irrigation, 100% Field Capacity (FC)) at (day/night) temperature of 23/17 ± 3 °C; T2, drought stress (60% FC); T3, heat stress (38 °C); T4, 10 mg·L^−1^ of SeNPs; T5, drought stress (60% FC) + 10 mg·L^−1^ of SeNPs; and T6, heat stress (38 °C) + 10 mg·L^−1^ of SeNPs.

As shown in Figure 1, 31-day-old wheat seedlings had been impacted by drought, heat stress, and SeNPs applications. Under drought and heat stress, wheat seedlings were weak, short, and yellowish compared with the control group. In addition, descriptive statistics for morphological traits of eight wheat genotypes grown under control (T1), drought stress (T2), heat stress (T3), 10 mg·L^−1^ of SeNPs (T4), drought stress + 10 mg·L^−1^ of SeNPs (T5), and heat stress + 10 mg·L^−1^ of SeNPs (T6) are presented in Table S3. Overall, morpho-physiological traits such as PH, SFW, SDW, RFW, and RDW of wheat genotypes grown under different conditions in the current experiment ranged from 25.37–51.51 cm, 3.29–5.15 g, 0.50–1.97 g, 0.72–4.21 g, and 0.11–1.23 g, respectively, as shown in Table S3.

**Figure 2:**
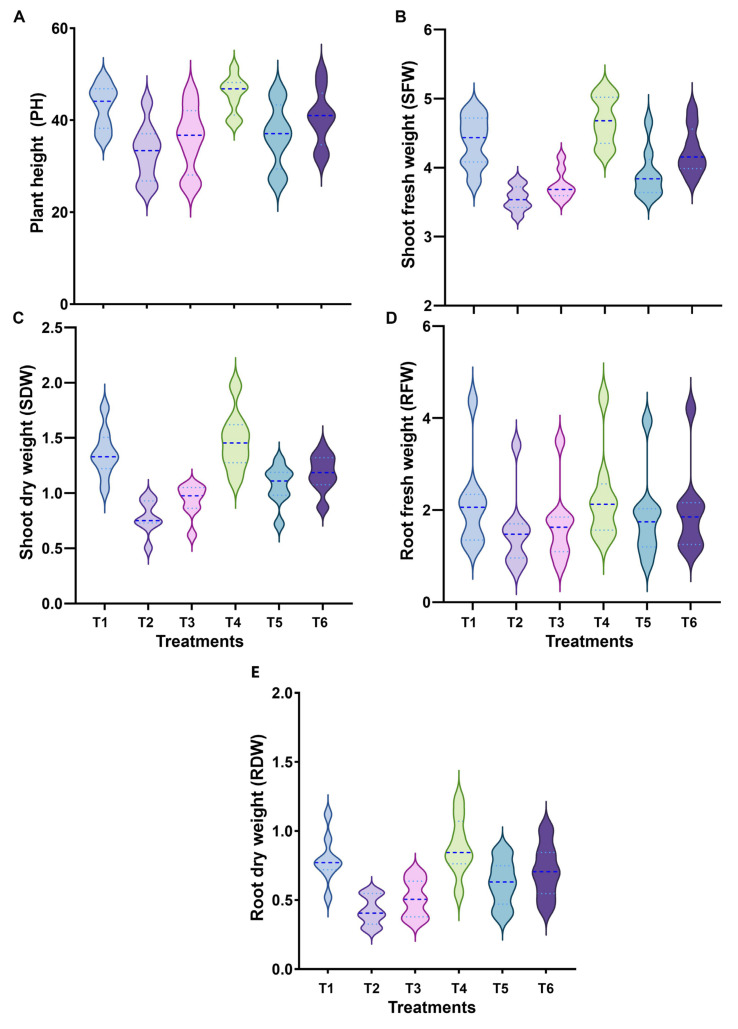
Comparison of morphological traits by violin plots: (**A**) plant height (PH), (**B**) shoot fresh weight (SFW), (**C**) shoot dry weight (SDW), (**D**) root fresh weight RFW), and (**E**) root dry weight (RDW) among eight wheat genotypes under drought, and heat stress and their response to SeNPs application. Treatments: T1, control (regular irrigation, 100% Field Capacity (FC)) at (day/night) temperature of 23/17 ± 3 °C; T2, drought stress (60% FC); T3, heat stress (38 °C); T4, 10 mg·L^−1^ of SeNPs; T5, drought stress (60% FC) + 10 mg·L^−1^ of SeNPs; and T6, heat stress (38 °C) + 10 mg·L^−1^ of SeNPs.

The performance of eight wheat genotypes on morphological traits grown under control, drought, and heat stress conditions without and with SeNPs foliar application was scored (Table S4). The results revealed a maximum significant reduction in plant height (PH) by 25.37 cm (Giza-171), shoot fresh weight (SFW) by 3.29 g (Misr-3), shoot dry weight (SDW) by 0.50 g (Sids-1), root fresh weight (RFW) by 0.72 g (Giza-168), and root dry weight (RDW) by 0.11 g (Shandweel-1) under drought stress compared with the control wheat plants. However, heat stress caused a maximum reduction of PH by 25.80 cm (Giza-171), SFW by 3.53 g (Giza-168), SDW by 0.62 g (Sids-1), RFW by 0.73 g (Giza-171), and RDW by 0.20 g (Shandweel-1) compared with the control wheat plants. We observed that Giza-168 and Sids-12 were the genotypes least affected by 2 stresses compared to the control wheat plants (Figure 2A–E and Table S4).

Foliar application of 10 mg L^−1^ of SeNPs significantly reduced the negative impacts of drought and heat stress on seedling growth parameters of wheat genotypes, where Sids-1 and Sids-12 recorded the highest responses (51.51 cm and 47.31 cm) to SeNPs for PH under drought and heat stress, respectively (Figure 2A). In the Giza-168 wheat genotype, SFW and RFW were the most impacted by SeNPs application. For shoot and root dry weight parameters, the greatest values of SDW and RDW of wheat plants grown under drought and heat stress were recorded in Misr-3 (1.30 g and 1.39 g) and Giza 171 (0.86 g and 1.02 g), respectively, and that was supplemented with SeNPs (Figure 2C,E and Table S4).

### 3.3. Physiological Responses of Wheat Genotypes to SeNPs

#### 3.3.1. Leaf Chlorophyll Content and Photosynthetic Rate

**Figure 3:**
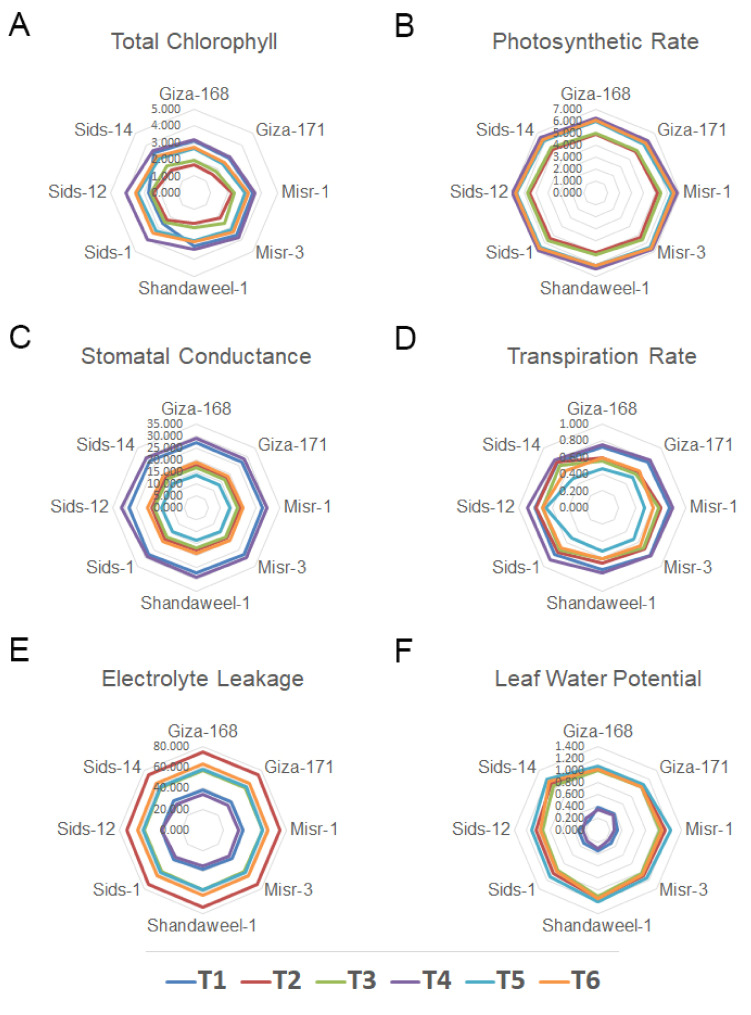
Radar analysis shows eight wheat genotypes’ performances for chlorophyll content, relative water relations, and gas exchange parameters under drought and heat stress and their responses to SeNPs application. (**A**) Total chlorophyll (mg·g^−1^ FW), (**B**) photosynthetic rate (μmol (CO_2_) m^−2^ s^−1^), (**C**) stomatal conductance (mmol H_2_O m^−2^·s^−1^), (**D**) transpiration rate (mmol H_2_O m^−2^·s^−2^), (**E**) electrolyte leakage (%), and (**F**) leaf water potential (MPa). Treatments: T1, control (regular irrigation, 100% field capacity (FC)) at (day/night) temperatures of 23/17 ± 3 °C; T2, drought stress (60% FC); T3, heat stress (38 °C); T4, 10 mg·L^−1^ of SeNPs; T5, drought stress (60% FC) + 10 mg·L^−1^ of SeNPs; and T6, heat stress (38 °C) + 10 mg·L^−1^ of SeNPs.

The effect of SeNPs on leaf chlorophyll content (CHLO) and photosynthetic rate (PN) for eight wheat genotypes under drought and heat stress conditions was investigated. As shown in Figure 3A,B and Tables S3 and S7, all the genotypes differ in their response to both stresses. The obtained results indicated that the Sids-12 genotype had the highest chlorophyll content and photosynthetic rates (2.45 mg·g^−1^. FW and 5.52 μmol (CO_2_) m^−2^·s^−1^), respectively, under drought stress, while it was at 2.90 mg·g^−1^. FW and 5.72 μmol (CO_2_) m^−2^·s^−1^, respectively, under heat stress, as compared to the control wheat plants (Figure 3A,B). However, the lowest leaf chlorophyll content and photosynthetic rates (1.56 mg·g^−1^ FW and 4.80 μmol (CO_2_) m^−2^·s^−1^), respectively, under drought stress were reported in the Giza-171 genotype, while under heat stress they were 1.84 mg·g^−1^ FW and 5.02 μmol (CO_2_) m^−2^·s^−1^, respectively, as compared to the control. Under regular irrigation conditions (100% FC) and foliar application of 10 mg·L^−1^ of SeNPs, leaf chlorophyll content and photosynthetic rate percentage were increased in all wheat genotypes. The maximum percentage of chlorophyll content (4.12 mg·g^−1^ FW) and photosynthetic rate (6.98 μmol (CO_2_) m^−2^·s^−1^) were recorded in Sids-12 genotypes, respectively, due to foliar application of SeNPs. Under drought and heat stress, spraying with SeNPs (10 mg·L^−1^) increased leaf chlorophyll content to the maximum percentage (3.35 and 3.45 mg·g^−1^ FW) in the Sids-12 and Sids-1 genotypes, respectively (Figure 3A,B). Meanwhile, the greatest percentages of photosynthetic rate (6.71 and 6.73 μmol (CO_2_) m^−2^·s^−1^) were recorded in the Sids-12 genotype sprayed with SeNPs and drought or heat stress, respectively, compared to wheat plants treated with drought or heat stress only (Figure 3A,B).

#### 3.3.2. Stomatal Conductance, Transpiration Rate, Leaf Electrolyte Leakage, and Leaf Water Potential

The indicators of stomatal conductance (SC) and transpiration rate (TR) of wheat seedlings were significantly reduced when wheat plants were treated with drought and heat stress either alone or in combination with SeNPs compared to the control wheat plants (Figure 3C,D and Tables S3 and S7).

The results demonstrated that the lowest decreasing values (18.61 and 0.79 mmol H_2_O·m^−2^·s^−2^) of SC and TR for wheat seedlings treated by drought stress alone were recorded in Misr-1 and Giza-168 genotypes, respectively, compared to the control wheat plants, demonstrating their tolerance/resistance to drought stress. However, the highest decreasing values (11.26 and 0.60 mmol H_2_O·m^−2^·s^−2^) were found in the Sids-1 and Sids-12 genotypes, respectively. Regarding heat stress, the lowest decreasing values (18.01 and 0.74 mmol H_2_O·m^−2^·s^−2^) of SC and TR appeared in the Sids-12 genotype, reflecting their resistance to heat stress, and the highest decreasing values (16.51 and 0.55 mmol H_2_O·m^−2^·s^−2^) observed in the Sids-12 and Giza-168 genotypes treated by heat stress alone compared to the control wheat plants (Figure 3C,D).

Under the full-irrigation condition (100% FC), the application of SeNPs increased stomatal conductance and transpiration rate in all wheat genotypes, where the maximum percentage value of SC (28.30 mmol H_2_O·m^−2^·s^−2^) and TR (0.81 mmol H_2_O·m^−2^·s^−2^) was recorded in the Sids-12 genotype. On the other hand, treatment with SeNPs (10 mg·L^−1^) in drought stress decreased the SC percentage in all wheat genotypes except Sids-12, where the SC percentage increased by 14.16% compared to drought stress control. However, the SC percentage increased in all studied wheat genotypes treated by heat stress with SeNPs except the Shandaweel-1 genotype, and the maximum percentage value of SC (20.30 mmol H_2_O·m^−2^·s^−2^) was reported in the Sids-12 genotype compared to heat stress control (Figure 3C,D).

Individual drought stress resulted in more electrolyte leakage (EL) and leaf water potential (LWP) as compared to individual heat stress and the control (Figure 3E,F and Tables S5 and S7). However, the percentage of EL differed significantly among various treatments and wheat genotypes. The lowest increased percentage of EL (73.15% and 37.30%) was observed in Sids-12 and Giza-171 genotypes treated by drought and heat stress, respectively, compared to the control treatment (Figure 3E). In contrast, treating wheat seedlings with SeNPs alone significantly decreased EL compared to the control wheat plants. For the interaction between drought and heat stress and SeNPs, our results indicated the ameliorative impact of SeNPs on two adverse stresses. Thus, a remarkable decrease was observed in seedlings treated with drought and SeNPs as compared to drought-control wheat plants. In contrast, the maximum reduction percentage (21.53%) in EL was found in the Sids-1 genotype compared to drought-treated wheat plants. The percentage of EL differed in wheat genotypes due to the interaction between heat stress and SeNPs, in which EL was increased in five genotypes and decreased in three genotypes. However, the highest percentage (63.30%) was observed in the Giza-171 genotype, and the lowest increase percentage (62.14%) was reported in Sids-12 compared with heat-treated wheat plants (Figure 3E).

The highest increases in LWP (1.15 and 1.11 MPa) were observed in the Shandaweel-1 genotype under drought and heat stresses, respectively; however, the lowest increases in LWP (0.15 and 0.92 MPa) were found in the Misr-3 and Sids-12 genotypes as compared to the control wheat plants (Figure 3F). Notably, decreases were observed in the LWP value for wheat seedlings treated by SeNPs singly compared to the control wheat plants, and the maximum decrease value (0.25 MPa) was recorded in the Misr-3 and Sids-12 genotypes. Moreover, markedly increased LWP values were observed in seedlings treated with drought + SeNPs or heat + SeNPs as compared to drought or heated stress wheat plants. In contrast, the value of the maximum increase (1.20 and 1.18 MPa) was observed in the Sids-14 genotype compared to drought and heat-treated wheat plants. However, the lowest increased value (1.05 and 0.97 MPa) of LWP value was found in Giza-168 and Sids-12 genotypes compared to drought or heat stress wheat plants (Figure 3F and Table S5).

### 3.4. Biochemical Responses of Wheat Genotypes to SeNPs under Drought and Heat Stress

#### 3.4.1. Leaf Malondialdehyde (MDA), Hydrogen Peroxide (H_2_O_2_), and Proline Contents

The content of MDA increased with drought and heat stresses compared with the control wheat plants (Tables S6 and S7). The results showed that under drought and heat stress, the oxidative damage to the leaves in the Giza-168 and Giza-171 genotypes was greater than that in the other studied genotypes. However, the Sids-12 genotype’s MDA content showed the lowest value among wheat genotypes, reflecting their tolerance to drought and heat stress. It also showed that the MDA content for all genotypes that were sprayed with 10 mg·L^−1^ of SeNPs resembled its content compared to spraying with water, and the greatest value was observed in Shandaweel-1 and the lowest one was noticed in Giza-168 compared to other genotypes (Table S6). Comparing the SeNPs sprayed and the control wheat plants under drought and heat stresses, the results revealed that spraying with SeNPs significantly increased MDA content by a percentage ranging from 0.86% to 17.14%. Our results also showed that the highest increase (4.87% and 17.14%) was recorded in Sids-14 and Misr-1 seedlings sprayed with 10 mg·L^−1^ of SeNPs under drought and heat stress, respectively, concerning their respective controls. Meanwhile, Giza-168 and Giza-171 recorded the lowest percentage of increase for MDA content (Table S6).

The content of H_2_O_2_ significantly increased with drought and heat stresses compared with the control wheat plants, while the content of H_2_O_2_ significantly decreased with spraying wheat seedlings with 10 mg·L^−1^ of SeNPs. In addition, H_2_O_2_ content increased dramatically at a greater rate in Giza-168 and Giza-171 seedling leaves than in other wheat genotypes under drought and heat stress with respect to their respective controls, indicating their sensitivity to both stresses (Table S6).

The proline concentrations in the leaves of wheat plants were significantly affected under drought and heat stress (Table S6). The leaf proline amount increased with drought stress (Table S6). Under drought stress, Giza-171 recorded the highest amounts of proline, with a 45.40% increase compared with the control wheat plants. Under drought stress, spraying the wheat plants with 10 mg·L^−1^ of SeNPs significantly increased the values of proline in most wheat genotypes compared to the control. The proline contents increased under heat stress, with the highest increase (a 50.55% increase) in the Giza-168 wheat genotype compared with the control.

#### 3.4.2. Antioxidant Enzymes: Catalase (CAT), Ascorbic Acid Peroxidase (APX), and Superoxide Dismutase (SOD)

Compared to the control treatment, the imposed stressors with and without SeNPs spraying increased SOD activity. SOD activity rose considerably with stress exposure, with the maximum SOD activity recorded under drought and heat stresses in the Sids-14 wheat genotype. In the control treatment, the Giza-168 genotype had the lowest SOD activity (Tables S6 and S7). It was also shown that spraying wheat seedlings with 10 mg·L^−1^ of SeNPs enhanced SOD activity for all genotypes as compared to spraying with water, with Sids-12 having the highest value and Giza-168 having the lowest value.

Drought raised ascorbic acid peroxidase (APX) activity more than heat stress in all wheat genotypes (Table S6). The highest APX activity was recorded in the Shandaweel-1 cultivar under drought stress as compared to the control (Table S6). Heat stress increased ascorbate peroxidase activity in the Giza-171 genotype compared to the control. The imposed stresses increased the CAT activity as compared to the control treatment (Table S6). The highest CAT activity was measured under drought and heat stresses in the Giza-171 wheat genotype as compared to the control. Wheat plants growing under drought and heat stress combined with 10 mg·L^−1^ of SeNPs did not show a significant difference in CAT activity.

### 3.5. Correlations Matrix of Morpho-Physiological and Biochemical Traits of Eight Wheat Genotypes to SeNPs under Drought and Heat Stress

**Figure 4:**
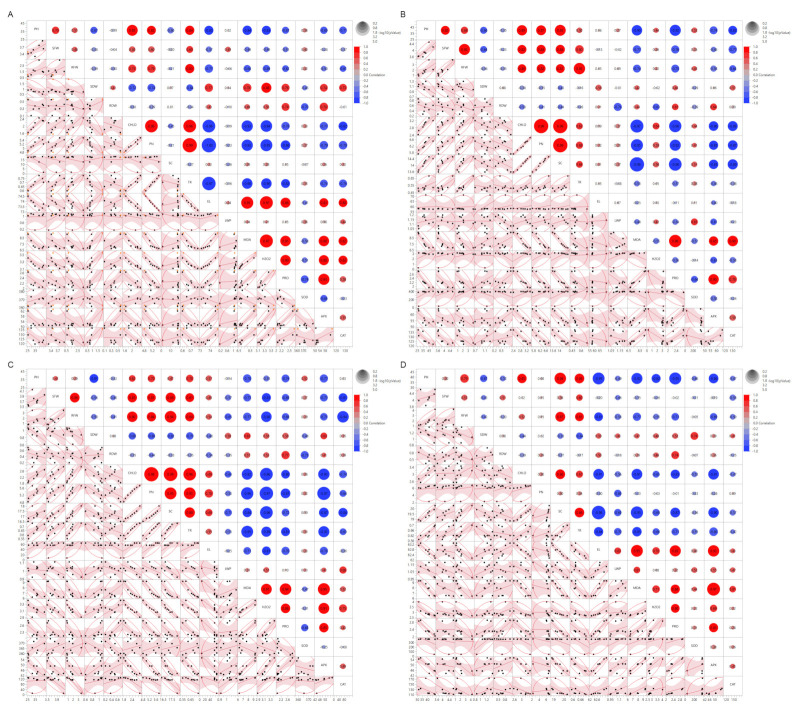
Scatter plot and heatmap correlation matrix of different morpho-physiological parameters for eight genotypes grown under (**A**) drought stress and (**B**) drought + SeNPs, (**C**) heat stress, and (**D**) heat + SeNPs. Abbreviations: Plant Height (PH), Shoot Fresh Weight (SFW), Shoot Dry Weight (SDW), Root Fresh Weight (RFW), Root Dry Weight (RDW), Total Chlorophyll (CHLO), Net Photosynthetic Rate (PN), Stomatal Conductance (SC), Transpiration Rate (TR), Electrolyte Leakage (EL), Leaf Water Potential (LWP), Malondialdehyde (MDA), Hydrogen Peroxide (H_2_O_2_), Proline (PRO), Superoxide Dismutase (SOD), Ascorbate Peroxidase (APX), and Catalase (CAT).

The scatter plot and heatmap correlation matrix revealed that PH is strongly associated with SFW, RFW, CHLO, PN, and TR. At the same time, it negatively correlated with RDW, SDW, MDA, H_2_O_2_, proline, APX, and CAT under drought stress. Likewise, SFW and RFW positively correlated with CHLO, PN, TR, and SOD, whereas they were negatively correlated with SDW, RDW, SC, EL, MDA, H_2_O_2_, proline, APX, and CAT (Figure 4A and Table S8). Likewise, SDW and RDW were positively correlated with EL, MDA, H_2_O_2_, proline, and APX, while negatively correlated with CHLO, PN, TR, and SOD. From physiological and water-relation parameters, CHLO and PN had a strong positive correlation with TR and SOD but were negatively correlated with SC, EL, LWP, MDA, H_2_O_2_, proline, APX, and CAT. Furthermore, SC and EL exhibited a negative correlation to TR, LWP, and SOD, whereas they were positively correlated to MDA, H_2_O_2_, proline, APX, and CAT. The MDA, H_2_O_2,_ and proline were strongly positive correlated to each other and with some enzymatic antioxidants such as APX and CAT. At the same time, they were negatively linked to changes in SOD.

Under drought stress and after SeNPs application, the morpho-physiological traits showed changes, such as PH and SFW becoming positively correlated with SC (Figure 4B and Table S9). Additionally, SFW had an increasingly positive correlation with RFW, CHLO, PN, and TR, whereas RFW had a decreasingly positive correlation with EL and LWP. Moreover, SDW showed an increasingly positive correlation with RDW and APX and converted to a positive correlation with H_2_O_2_ and CAT. In contrast, it is negatively associated with SC, MDA, and proline. Furthermore, CHLO and PN converted to a strongly positive correlation with SC and H_2_O_2,_ and they had a reduction in the positive correlation with TR. In addition, SC had a positive correlation with TR and LWP, and it converted to a strongly negative correlation with MDA and proline. In the same trend, TR converted to a moderately positive correlation with EL and H_2_O_2_ and reduced its negative correlation with MDA and proline. Additionally, EL converted to a negative correlation with MDA, proline, CAT, and APX, while it had a reduction in its positive correlation with H_2_O_2_. Moreover, LWP exhibited a negative correlation with MDA and proline and converted to a negative correlation with APX and CAT. Finally, there was a significant variation in which MDA became negatively correlated to H_2_O_2_ and showed an increasingly positive correlation with proline, whereas H_2_O_2_ became negatively correlated to proline, APX, and CAT (Figure 4B and Table S9).

Under heat stress, PH exhibited a high relationship with SFW, RFW, CHLO, PN, SC, and TR, whereas it was negatively correlated with RDW, SDW, MDA, H_2_O_2_, proline, and APX (Figure 4C and Table S10). Similarly, SFW and RFW were positively connected with CHLO, PN, TR, EL, and SOD but were negatively correlated with SDW, RDW, MDA, H_2_O_2_, proline, APX, and CAT. Similarly, SDW and RDW were shown to be favorably related to MDA, H_2_O_2_, proline, CAT, and APX, while negatively related to CHLO, PN, TR, SC, and SOD. Furthermore, CHLO and PN exhibited a substantial positive connection with SC, EL, TR, and SOD but were negatively correlated with LWP, MDA, H_2_O_2_, and proline. Additionally, SC and TR were shown to have a negative association with LWP, MDA, H_2_O_2_, proline, APX, and CAT but a positive correlation with EL and SOD. MDA, H_2_O_2_, and proline were positively connected with each other and with several enzymatic antioxidants such as APX and CAT. In contrast, they were negatively related to changes in SOD (Figure 4C and Table S10).

When compared to parameters under heat stress and after SeNPs application, the morpho-physiological features changed, with PH being adversely correlated with EL and somewhat negatively correlated with SOD (Figure 4D and Table S11). SFW also exhibited a lower positive correlation with CHLO, PN, SC, and TR, whereas RFW had a lower positive association with PN. Furthermore, SDW was favorably associated with EL, whereas RDW was negatively associated with SOD and CAT and positively associated with PN and EL. Likewise, CHLO lost its positive association with SOD, while PN dropped its positive correlation with SC and TR. SC also exhibited a negative correlation with EL and SOD. TR converted to a significant negative relationship with EL along the same lines. In addition, EL developed a positive correlation with LWP, MDA, H_2_O_2_, proline, CAT, and APX. MDA, H_2_O_2_, and proline interacted favorably with SOD, whereas SOD became positively associated with APX and CAT (Figure 4D and Table S11).

### 3.6. Expression Level of Stress-Responsive Genes in Response to Drought and Heat Stress Conditions

**Figure 5:**
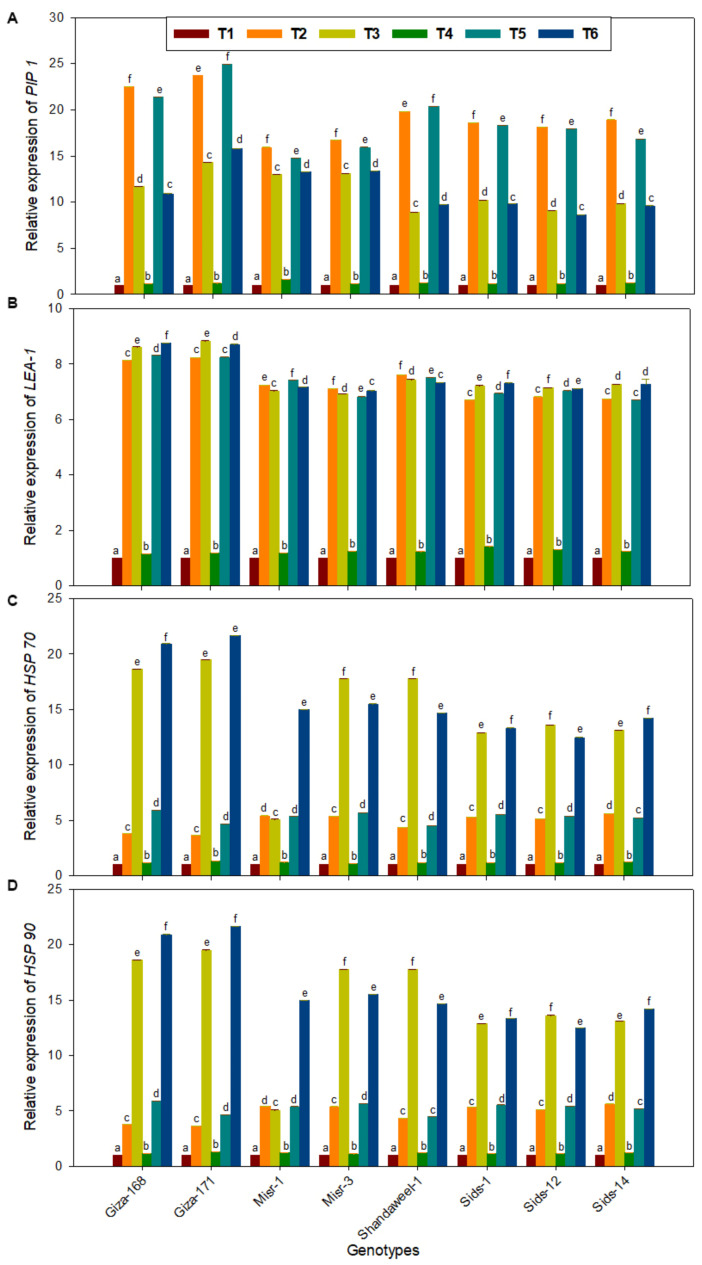
Expression of stress-responsive genes (**A**) *PIP1*, (**B**) *LEA-1*, (**C**) *HSP 70*, and (**D**) *HSP 90* of eight wheat genotypes grown under drought and heat stress and their response to SeNPs application. Different letters above the columns indicate statistically significant differences among treatments according to (Tukey’s HSD post hoc test) at *p* ≤ 0.05. Treatments: T1, control (regular irrigation, 100% field capacity (FC)) at (day/night) temperatures of 23/17 ± 3 °C; T2, drought stress (60% FC); T3, heat stress (38 °C); T4, 10 mg·L^−1^ of SeNPs; T5, drought stress (60% FC) + 10 mg·L^−1^ of SeNPs; and T6, heat stress (38 °C) + 10 mg·L^−1^ of SeNPs.

Under drought and heat stress, qRT-PCR analysis of four stress-responsive genes was conducted to verify our results for morphological, physiological, and biochemical analysis. Those genes were late embryogenesis abundant (*LEA-1*), aquaporin (*PIP1*), heat shock protein 70 (*Hsp70*), and heat shock protein 90 (*Hsp90*) genes. They were conducted on 8 wheat genotype seedlings at 31 DAS under drought and heat stresses in the presence or absence of SeNPs (Figure 5).

The results of qRT-PCR showed that different stress treatments differentially induced the expression of these four stress-responsive genes. The gene expression profiling data were also found to be considerably in correlation with our physiological and biochemical results under these two stresses (Figure 5).

Most significantly, drought stress induced the expression of the *PIP1* gene in all wheat genotypes leaves (Figure 5A). The highest *PIP1* gene expression was recorded in Giza-171, followed by Giza-168. In addition, *LEA-1* gene expression was significantly upregulated in all wheat genotypes under drought and heat stressors with and without SeNPs. Giza-168 and Giza-171 showed the highest upregulation of the *LEA-1* gene under two stresses with and without SeNPs, whereas Misr-1 and Misr-3 had the highest downregulation (Figure 5B).

Under heat stress with and without SeNPs, both Giza-168 and Giza-171 showed the highest upregulation of *Hsp70* and *Hsp90* genes, whereas Misr-1 and Misr-3 had the lowest gene upregulation (Figure 5C,D). In drought and heat stress seedlings, expression of *LEA-1* in stressed wheat plants increased 7–8-fold compared to the control. In the case of drought with SeNPs-treated wheat seedlings, maximum expression of *PIP1* was observed at the Giza-171 wheat genotype (25-fold) compared to the control.

### 3.7. Biplot among the Morpho-Physiological Traits and Stress Responsive Genes

**Figure 6:**
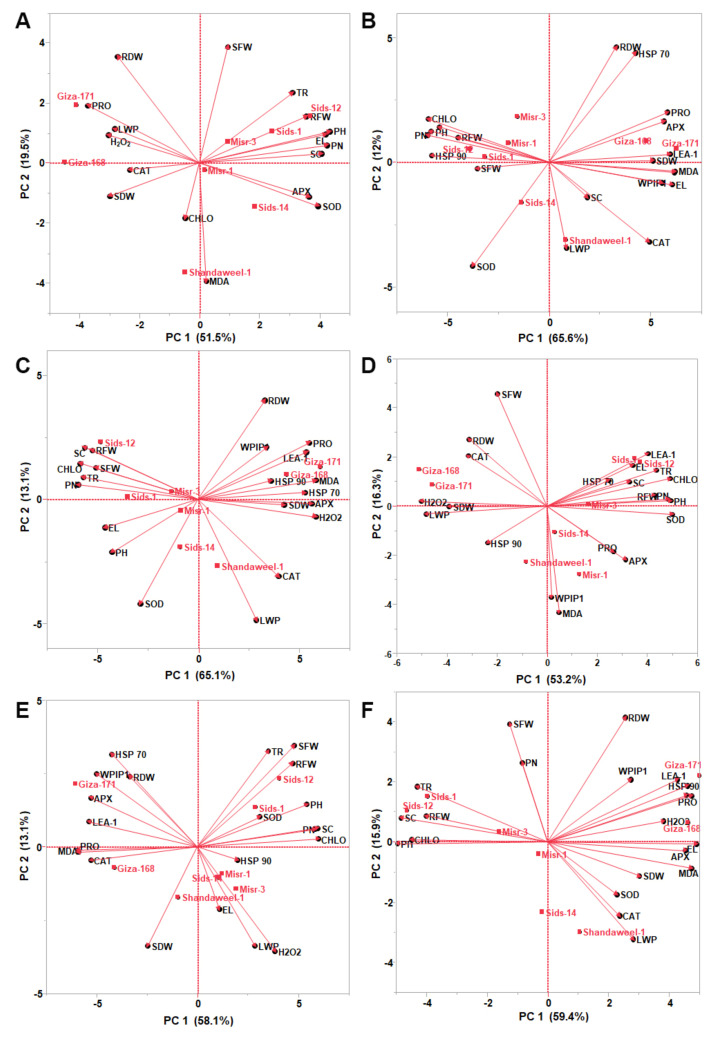
Distribution of eight wheat genotypes on biplot and the score plot PC1 and PC2 under different conditions: (**A**) Control (regular irrigation, 100% FC) (T1), (**B**) Drought (60% FC) (T2), (**C**) Heat (38 °C) (T3), (**D**) 10 mg·L^−1^ of SeNPs (T4), (**E**) Drought + 10 mg·L^−1^ of SeNPs (T5), and (**F**) Heat + 10 mg·L^−1^ of SeNPs (T6). Abbreviations: Plant Height (PH), Shoot Fresh Weight (SFW), Shoot Dry Weight (SDW), Root Fresh Weight (RFW), Root Dry Weight (RDW), Total Chlorophyll (CHLO), Net Photosynthetic rate (PN), Stomatal Conductance (SC), Transpiration Rate (TR), Electrolyte Leakage (EL), Leaf Water Potential (LWP), Malondialdehyde (MDA), Hydrogen Peroxide (H_2_O_2_), Proline (PRO), Superoxide Dismutase (SOD), Ascorbate Peroxidase (APX), and Catalase (CAT).

A PCA-biplot was used to analyze the average data of morpho-physiological traits and genomic constitution. PCA reflects the significance of the most significant contributor to total variation along each differentiation axis. PCA revealed similar groups, correlating the results of the interrelationship among all measured morpho-physiological and molecular parameters under different conditions (Figure 6).

Under regular irrigation (control), variation in the eight-wheat genotype described by the first two PCs per cluster ranged from 19.5 to 51.5% of the total variation. In the system of the first two components, the length of the vector and cosine of the angle were used to discriminate wheat genotypes (Figure 6A). The eight studied genotypes were also grouped into three clusters: cluster I included five genotypes (Misr-1, Misr-3, Sids-1, Sids-12, and Sids-14); cluster II had two genotypes (Giza-168 and Giza-171); and cluster III had only one genotype (Shandweel-1), which was distinct and well supported. Furthermore, objects with similar ordinates were more alike than those with different ordinates. Cluster I had five genotypes with frequent vectors such as PH, RFW, and PN, which had a strong positive correlation with each other. Additionally, SOD and APX had strong discriminating power between Sids-14 and Misr-1. Moreover, SOD created 90 degrees with SFW; the same pattern was also shown between LWP and SFW, in which they were not likely to be correlated. Proline, LWP, and H_2_O_2_ are the most extended vectors, and the small angle between these vectors proves a significant and strong positive correlation between these traits in cluster II. On the other side, SOD and APX traits had a strong positive correlation with each other but had an extremely negative correlation with proline, LWP, and H_2_O_2_. Moreover, the vector for MDA showed the strong discrimination power of the Shandweel-1 genotype among the other wheat genotypes (Figure 6A).

Under drought conditions, PCA1 described 65.6%, while PCA2 reported 12% of the total variation (Figure 6B). Biplot categorized the studied genotypes into three clusters. Cluster I had two genotypes (Giza-168 and Giza-171) with frequent vectors such as *LEA-1*, SDW, EL, and SDW. Additionally, *HSP70* and RDW had a strong positive correlation with each other, whereas *HSP70* and SOD had larger cosines of the angle with which they were not likely to be correlated. Cluster II had four genotypes (Misr-1, Misr-3, Sids-1, and Sids-12), with frequent vectors such as *HSP90*, PH, TR, RFW, and SFW that had a strong positive correlation with each other. LWP and CAT were the most frequent vectors to differentiate cluster III (Sids-14 and Shandweel-1) genotypes from the others (Figure 6B).

Under heat conditions, PCA1 reported 65.1%, while PCA2 described 13.1% of the total variation (Figure 6C). Biplot grouped the studied genotypes into three clusters. Cluster I had two genotypes (Giza-168 and Giza-171) with frequent vectors such as MDA, H_2_O_2_, proline, APX, *HSP90*, and *PIP1*. In addition, cluster II had three genotypes (Sids-1, Sids-12, and Misr-3) with strong vectors such as CHLO, PN, SC, RFW, and SFW. EL and PH vectors had strong discrimination power between clusters II and III, which had three genotypes (Misr-1, Sids-14, and Shandweel-1). The most frequent vector, SOD, which differentiates within cluster III genotypes, had a negative correlation with RDW (Figure 6C).

With SeNPs spraying, the variation of the studied genotype described by the first two PCs per cluster ranged from 16.3 to 53.2% of the total variation, as illustrated in Figure 6D. Biplot is categorized into three clusters in the same drought condition pattern. Cluster I had Sids-1, Sids-12, and Misr-3 genotypes with frequent vectors such as *LEA-1*, PH, TR, El, SC, and RFW with strong positive correlations among them. In contrast, cluster II had Giza-168 and Giza-171 genotypes with frequent vectors such as H_2_O_2_, LWP, and SDW. Cluster III had three genotypes (Misr-1, Sids-14, and Shandweel-1), which differentiated from others by MDA, *PIP1*, APX, and proline (Figure 6D).

Under drought and SeNPs conditions, PCA1 reported 58.1% of the total variation, while PCA2 described a 13.1% (Figure 6E). The three clusters were categorized as follows: cluster I had two genotypes (Sids-1 and Sids-12) with PH, CHLO, SFW, and TR strongly positive correlated vectors; cluster II also had two genotypes (Giza-168 and Giza-171) with MDA, CAT, APX, *PIP1*, *LEA-1*, and *HSP70* vectors; and the cluster III had Misr-1, Misr-3, Sids-14, and Shandweel-1 with discriminating vectors (H_2_O_2_, LWP, LE, and SDW) as described in Figure 6E.

Under heat and SeNPs conditions, PCA1 reported 59.4%, while PCA2 described 15.9% of the total variation (Figure 6F). The three clusters were categorized as follows: cluster I had two genotypes (Giza-168 and Giza-171) with *HSP70*, *HSP90*, *PIP1*, *LEA-1*, H_2_O_2_, EL, APX, and MDA vectors; cluster II had four genotypes (Sids-1, Sids-12, Misr-1, and Misr-3) with SC, TR, PH, CHLO, and RFW vectors; and cluster III had two genotypes (Sids-14 and Shandweel-1) with LWP, CAT, and SOD vectors, which strongly positive correlated as observed in Figure 6F.

## 4. Discussion

According to current climate estimates, drought and heat waves are expected to become more prevalent and extreme in the future [35]. Every degree Celsius increase in the global average temperature reduces the global wheat yield by 6.0% [36]. Drought and heat stress can lower organ size (leaf, tiller, and spikes) and growth period at different phases of development (tillering, jointing, booting, heading, anthesis, and grain filling) [37]. Lack of water and nutrients contribute to a reduction in plant development and production [38]. A study found that the plant grown with the stimulation of selenium nanoparticles was 50% higher than the control option [39].

A range of strategies is being utilized to increase plant abiotic stress tolerance, including NPs, which are expected to boost efficiency under stressful circumstances. NPs improved germination and seedling development, physiological and biochemical activities such as photosynthesis and nitrogen metabolism, chlorophyll quality, antioxidant enzyme activities, carbohydrate and protein content, and positive gene expression, implying their potential use in crop enhancement [40]. According to Germ et al. [41], applying selenium at low concentrations has favorable benefits and boosts plants’ ability to withstand drought stress. Furthermore, other researchers found that trace selenium concentrations improve plant development, postpone senescence, and control plant water levels under water-stressed settings [42]. The negative impact was more severe under drought conditions than under heat stress conditions. A low-dose of SeNPs (10 mg·L^−1^) improved all seedling development metrics (PH, RFW, RDW, SFW, and SDW) in wheat types when compared to negative control circumstances (drought and heat stress) and normal watering (100% FC). Seedling metrics improved more pronouncedly in Giza-168 and Sids-12 genotypes treated with 10 mg·L^−1^ of SeNPs. Our findings support the findings of Shoeibi et al. [43], who demonstrated that biogenic SeNPs increase organogenesis and root development in various plants. Foliar treatments of SeNPs increase wheat plants’ morphological characteristics under drought circumstances [44]. Treatment with nano-selenium resulted in an increase in antioxidants, photosynthetic rate, insect, and disease resistance [45]. Furthermore, these biogenic nanoparticles improve plant antioxidant defense under water stress conditions [46]. Furthermore, Ikram et al. [47] discovered that 30 mg·L^−1^ of SeNPs is more effective in boosting the development of wheat under drought conditions compared to the control.

Shoot length, root length, and fresh weight are considered essential criteria for choosing drought-tolerant wheat genotypes [48]. In wheat genotypes with variable shoot-root dry weight reduction, mechanisms such as osmoregulation, antioxidant defenses, and heat shock proteins can be found to be responsible for high temperature and drought tolerance [48]. Drought stress alters the osmotic potential of the cell, resulting in inefficient cell division and, as a result, a loss in root and shoot fresh weight [49]. This study showed a maximum decline in PH, SFW, SDW, RFW, and RDW under drought and heat stress. Compared to the control wheat plants, SFW and RFW dropped as stress intensity increased. Our findings were consistent with the findings of Shunkao et al. [50]. Moreover, Amoah et al. [51] reported that leaf dry weight (LDW) and RDW dropped up to 35.71% and 67.69%, respectively, in wheat genotypes under drought stress. Ahmed, et al. [52] discovered that the Chakwal-50 wheat genotype had the highest RDW, SDW, and SFW but the shortest PH.

The scatter plot and heatmap correlation matrix between morpho-physiological and biochemical parameters of eight wheat genotypes were used in this investigation. Plant height, RFW, SFW, RDW, and SDW were among the seedling morphological features evaluated. Understanding the relationship between these parameters was critical for improving the effectiveness of wheat drought tolerance breeding [53]. A correlation study revealed that PH had a close correlation with SFW and RFW but a negative relationship with RDW and SDW under stressful circumstances. While root length was favorably associated with root–shoot ratio, fresh weight, and dry weight, it was negatively connected to shoot length and relative water content in wheat plants [54].

Drought directly affects wheat on all yielding and yield-related traits, as the yield decline due to persistent drought stress (CD, 83.60%) was much greater than that due to terminal drought stress (TD, 26.43%) [55]. A temperature rise above 30 degrees Celsius leads to the death of pollen grains, the failure to fill the grain, and the failure to form the grain itself [56]. Drought inhibits shoot development while stimulating root growth and decreasing chlorophyll content [57]. Wheat plants enhance canopy temperature during heat and drought stress by rapidly shutting their stomata, resulting in less transpiration and water loss. Consequently, decreased stomatal opening lowers CO_2_ fixation, resulting in decreased photosynthesis and chlorophyll concentrations. Photosynthesis is also particularly sensitive under heat and drought circumstances, as the ratio and amount of chlorophyll (a and b) and carotenoid decreased with rising heat and drought severity [58]. Furthermore, the reduction in CHLO concentration in wheat leaves due to heat stress might be related to impaired CHLO production or faster breakdown [59]. In this study, induced stresses considerably impacted both leaf chlorophyll concentration and photosynthetic rates. Under drought stress, the Sids-12 genotype performed better regarding CHLO and PN, whereas the Giza-171 genotype performed the worst. Compared to wheat plants solely subjected to drought or heat stress, the Sids-12 genotype sprayed with SeNPs performed the best. Additionally, Mohi-Ud-Din et al. [60] revealed that wheat genotypes show improvement in extreme heat conditions. The value of chlorophyll levels is less than the wheat plant’s exposure to heat stress and drought. Previous studies reported that under drought stress, the drop in photosynthetic pigments, the chlorophyll concentration, ranged from 43% to 27.82% across wheat genotypes [51,61].

Plant tissue temperature is sustained via water intake and transpiration, which stabilizes the water content of the tissue. However, when there is water scarcity, a temperature rise might be fatal [62]. Leaf relative water content (LRWC), leaf water potential (LWP), rate of transpiration (TR), and stomatal conductance (SC) are all regulated by leaf and canopy temperature under heat stress. However, drought stress reduced the transpiration rate, whereas the combined impact reduced it by 60–63%. Accordingly, the effect of heat on transpiration and the effect of drought on transpiration as well lead to a significant loss in the processes of photosynthesis and metabolism within the plant. Furthermore, using SeNPs in conjunction with either drought or heat stress resulted in a significant decrease in TR values for most wheat genotypes, except for the Giza-168 and Giza-171 genotypes, which showed a minor rise in TR values when treated with heat stress and SeNPs. Our findings are consistent with the results of Qaseem et al. [38], who discovered that the rate of transpiration in the plant increased to some extent under the pressure of high temperatures compared to the control and that this led to the loss of water in the plant.

Our results supported the findings of Bezabeh et al. [61], who discovered genetic heterogeneity in high temperature-induced electrolyte leakage in wheat genotypes. Water scarcity in the cell stimulates leakage of electrolytes and peroxidation of lipids from the thylakoid membrane of the chloroplast, which leads to a loss of chlorophyll concentration and has a harmful effect on all other features [63]. In this investigation, PH and SFW correlated positively with SC after SeNPs treatment under drought stress. Furthermore, SFW exhibited a higher positive association with RFW, CHLO, PN, and TR, but RFW had a lower positive correlation with EL and LWP than their respective drought stress. Similarly, applying selenium and silicon dioxide nanoparticles increased photosynthetic pigments in stressed strawberry fruits. At 100 mg·L^−1^ concentration, the Se/SiO_2_ NPs improve plant properties such as relative water content (RWC), membrane stability index, and water usage efficacy [64].

Heat, salt, and drought stress all induce the creation of reactive oxygen species (ROS), which cause oxidative damage in the plant and, eventually, death. Under heat stress, ROS accumulate, causing severe oxidative damage to the plant and impeding growth and development [65]. To combat ROS toxicity, a highly effective antioxidant defense mechanism comprising both nonenzymatic and enzymatic elements is necessary [66]. As a defensive strategy, wheat plants developed enzymatic antioxidants (SOD, APX, POX, and CAT), proline, glycine betaine, and sugar [67].

A high MDA concentration in this study implies membrane lipid peroxidation, which might indicate the amount of damage under unfavorable conditions. MDA content increased much faster in Giza-168 and Giza-171 seedling leaves than in other wheat genotypes during drought and heat stress, indicating their susceptibility to both stressors. H_2_O_2_ is a hazardous molecule that harms cells by causing lipid peroxidation and membrane damage [68], culminating in the production of the highly reactive and cytotoxic aldehyde derivative MDA [68]. In this study, drought and heat stress significantly boosted H_2_O_2_ accumulation in wheat plants, resulting in greater H_2_O_2_ accumulation in wheat plants subjected to both conditions. As previously stated, the drought and heat stress-induced rise in H_2_O_2_ led to a significant increase in leaf electrolyte leakage EL and MDA. The use of nano-selenium affects and improves germination and is a strong seed, as the coefficients of stem length, root length, wet weight, and dry weight were improved by 22.8, 24.9, 19.2, and 20%, respectively, over untreated controls [69].

**Figure 7:**
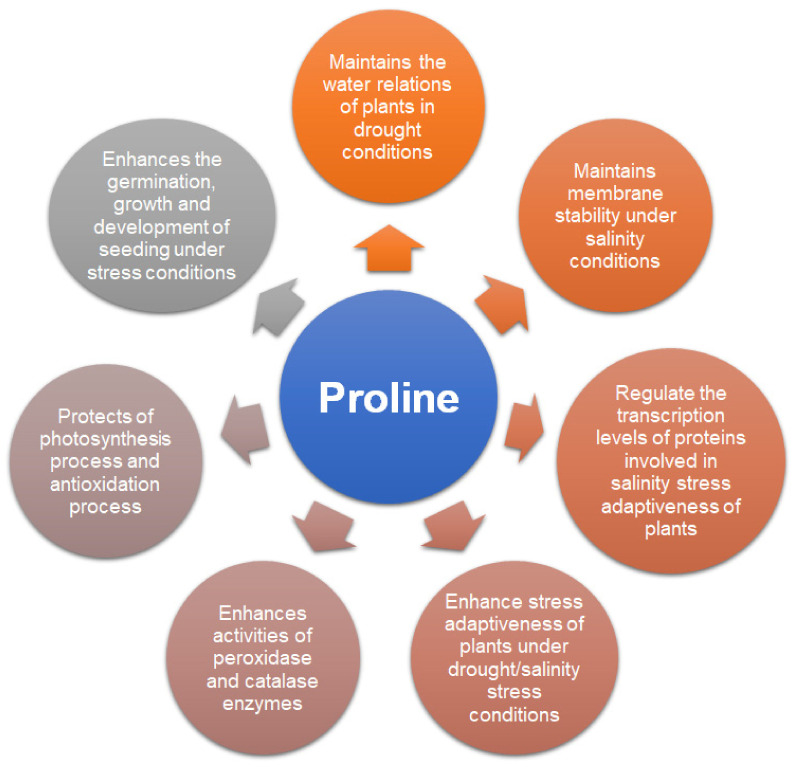
An overview of proline’s role in conferring abiotic stress tolerance in plants.

In response to abiotic stresses, wheat plants produce many types of osmolytes, including proline and soluble sugars, which enable them to tolerate stress through osmoregulation and ROS detoxification [70]. In the current study, both drought and heat stress increased proline content. When wheat plants were treated with 10 mg L^−1^ of SeNPs, proline concentrations decreased significantly, perhaps mitigating the deleterious effects of drought and heat stress. According to Amoah et al. [51], the maximum increase in EL, H_2_O_2_ accumulation, MDA, and proline content was 64.20%, 25.67%, 54.49%, and 14.49%, respectively, while the minimum increase in these parameters was 43.01%, 5.61%, 25.81%, and 5.6%, respectively, in wheat genotypes under drought stress. This is consistent with earlier research that has found even larger increases in heat-stressed wheat plants. In addition, Iqbal et al. [71] discovered that heat stress increases proline (8.2%) compared to the control. The application of AgNPs (50 mg·L^−1^) reduced the proline content (4%) of wheat plants’ resistance to heat stress. Although osmolytes increased in some AgNPs (50 mg·L^−1^) treated with heat stress, the total rise was less significant when compared to heat stress application alone. Osmolytes, such as proline, play a critical role in providing resistance against abiotic stress (Figure 7) [72].

Antioxidant enzymes such as SOD, CAT, and APX are all key antioxidant enzymes in the wheat genotypes in this study. SOD activity increased much more than CAT and APX in all sensitive and tolerant genotypes in response to stress. When wheat plants were treated with 10 mg·L^−1^ of SeNPs, there was a non-significant decrease in SOD, APX, and CAT activity. The results indicated that at 10 mg·L^−1^, only the control (T1) or SeNPs (T4) had reduced enzyme activity. Previously, increased POD and APX activities were found to operate as a mechanism of plant drought resistance [73]. Our findings showed that drought- and heat-tolerant “Sids-14” seedlings mitigated ROS generation by maximally increasing SOD activity under both stressors. Singh and Husen [74] reported that SeNPs can protect wheat plants with enhanced antioxidative defense systems under drought and heat stress. Furthermore, compared to the control, the stress-tolerant genotype Giza-171 showed the highest CAT activity under drought and heat stress and the highest APX activity under heat stress. Our results align with earlier research that found a considerable rise in SOD [75].

Drought decreases or eliminates chlorophyll concentration, which generates ROS such as O_2_ and H_2_O_2_, which can lead to lipid peroxidation [76]. Drought stress promotes electrolyte and lipid peroxidase leakage from the chloroplast’s thylakoid membrane, resulting in chlorophyll loss in plants [77]. There was a significant alteration in MDA, H_2_O_2_, and proline correlations after SeNPs administration to drought stressed wheat genotypes, with MDA becoming negatively associated with H_2_O_2_ and showing an increasingly positive correlation with proline, while H_2_O_2_ became negatively correlated to proline, APX, and CAT. After applying SeNPs to heat-challenged wheat genotypes, the MDA, H_2_O_2_, and proline interacted favorably with SOD, while SOD became positively connected with APX and CAT. When SeNPs are applied to wheat plants, they may cause acclimation reactions that lead to abiotic stress tolerance by up-regulating antioxidant enzymes. Although not yet recognized, seleniferous peptides and selenoamino acids are predicted to either directly neutralize ROS or do so through the production or modulation of other antioxidants in wheat plants [64]. Se/SiO_2_ NPs are known to increase drought tolerance in stressed plants by boosting the activity of antioxidant enzymes such as GPX, SOD, APX, and CAT, as well as lowering lipid peroxidation and H_2_O_2_ concentrations [78].

Non-enzymatic antioxidant systems comprise low-molecular-weight compounds such as vitamins (vitamins C and E), β-carotene, uric acid, glutathione, ascorbic acid, carotenoids, phenolic compounds, α-tocopherols, alkaloids, flavonoids, carbohydrates, oils, anthocyanin, proline, glycine-betaine, non-protein amino acids, and hormones [79].

Heat stress significantly influenced gene expression, but there were only minor variations in methylation patterns in a genome-wide analysis of DNA methylation in wheat. However, methylation was linked to minor alterations in gene expression of key genes during heat stress in certain circumstances [80]. These findings suggest that DNA methylation is linked to changes in heat stress response genes and warrant further investigation. The heat stress genes detected in wheat are primarily involved in primary and secondary metabolism, regulation, transcription, translation, and phytohormones, sugar, calcium, lipid signaling, or phosphorylation. Heat stress adaptations in wheat plants rely heavily on activating heat stress-sensitive transcription factors, heat shock proteins (HSPs), and ROS scavenging capacity [81]. The expression of *Hsp70* and *Hsp90* genes was elevated in this study when compared to the response to elevated temperature. Our findings are consistent with those of Bi et al. [82], who determined that gene expression profiling revealed that *TaHsfA2e-5D* was expressed consistently in numerous wheat tissues, most notably roots during the reproductive stage. Heat, cold, drought, high salinity, and various phytohormones increased the expression of *TaHsfA2e-5D* in wheat seedlings.

A number of the ABA-inducible genes were up-regulated by the stress encoded LEA proteins. *HVA1* (AT3G15670.1), a barley member of this protein group, has been demonstrated to be positively correlated with drought tolerance in barley and other plant species [82]. Another *LEA* gene reported to be drought-responsive is *WSI18* (AT3G15670.1) [83]. Wheat is a dynamic crop with multiple genes associated with drought stress. The LEA protein encoder is one of these *LEA* genes. Transcription factors alter shape when a wheat plant encounters water loss or scarcity. When exposed to drought stress, the *LEA* protein reacts to helicase, glucose, or rubisco [84]. Wheat contains at least 57 dehydrin genes and 429 *LEA* genes, whereas other plant species include around 15 dehydrin genes and 100 *LEA* genes [85]. Furthermore, MIPs (major intrinsic proteins) engage in the control of stomatal movement in a drought-treated plant’s leaf [86]. However, at the root, plasma membrane intrinsic proteins (PIPs), a subclass of the *MIP* family, are often reduced in abundance, so helping to limit water loss [87]. In our work, *PIP1* and *LEA-1* genes were considerably increased in response to drought stress, particularly *PIP1* genes. Drought and heat stress induced the expression pattern of most *LEA-1* genes under different conditions as evaluated by qRT-PCR analysis. The tolerant genotypes can modify genes under drought stress more strongly than the more vulnerable upland cotton genotypes [88].

Principal component analysis (PCA) is a multivariate statistical tool used to examine and simplify complicated and enormous datasets. Only PCAs with eigenvalues greater than one were considered significant. During heat conditions, PCA1 contributed 65.1% to the total variance and PCA2 contributed 13.1%. Xhulaj et al. [89] discovered that the three main components account for 66.42% of total data variance, with a comparable proportion of PCA1 and PCA2. Furthermore, Luković et al. [90] indicated that the first two axes of the biplot, PCA1 and PCA2, accounted for 57.31% of the total data variance of wheat under extremely wet circumstances. In this investigation, biplot classified the genotypes under both pressures into three groups. Under drought stress, the most common vectors discriminating across clusters were PH, SDW, SFW, RFW, EL, TR, LWP, CAT, *LEA-1*, and *HSP90*. The most common vectors under heat stress were RFW, SFW, CHLO, PN, SC, MDA, H_2_O_2_, proline, APX, SOD, *HSP90*, and *PIP1*. Our PCA results were comparable to those published by wheat researchers [51]. In this way, the traits studied are recognized as indicators of drought and heat tolerance for wheat varietal selection.

As a last point, selenium nanoparticles (SeNPs) increase the fraction of organic Se in wheat grain, which will improve the human body’s ability to absorb Se [91]. In this way, SeNPs are more environmentally friendly types of Se that can mitigate some of the drawbacks of regular Se supplements [92]. The results of this study show that SeNPs activate numerous genes and enhance their expression, leading to innovative approaches to cultivating plant cells and tissues, as well as applications for cultivation in terms of plant growth, yield, stress tolerance, and metabolism.

## 5. Conclusions

Drought and heat are the two significant environmental variables limiting agricultural yield worldwide. The primary scientific issues are improving drought and heat tolerance and eliminating the detrimental effects of stress in wheat. The current study concluded that foliar treatment with 10 mg·L^−1^ of SeNPs fortifies wheat crops against stress and improves morpho-physiological, biochemical, and molecular characteristics. It was found that four stress-responsive genes were significantly induced, suggesting that the ability of wheat plants to acclimate and induce antioxidant defenses may play a part in determining genotypic differences in drought and heat tolerance. Foliar application of SeNPs on wheat plants under stress enhances plant growth and maintains better water relations, reduces membrane damage, improves photosynthetic activity, and shows differentiation in gene expression trends. Outperforming drought and heat-tolerant wheat genotypes (Giza-168 and Giza-171) were followed by Sids-12 and Sids-14 wheat genotypes, which were able to maintain higher dry matter and pigment stability due to increased proline, lower accumulation of H_2_O_2_, MDA contents, and reduced oxidative stress with enhanced antioxidant capacity. The findings of the current study can be used in breeding programs to generate new promising wheat genotypes with improved drought and heat tolerance/resistance. In addition, our results provide a foundation for future molecular research in wheat at the seedling stage, modifying the antioxidant system’s related biochemical responses to better adapt wheat to early-stage stress. More research is needed to construct a pathway for converting data from the seedling stage to the other developmental phases, including yield and related characteristics. Several bioactive and biosafety features have been identified in SeNPs. To use SeNPs commercially, it is necessary to identify any negative effects they may have on agriculture and ecosystems.

## Figures and Tables

**Table 1:** Experimental design and treatments of eight wheat genotypes under drought and heat stresses without and with SeNPs foliar application.

Treatments	Experiment Time Course		
	0–7 DAS	7–21 DAS	21–31 DAS (SeNPs Application)
T1	Regular irrigation (100% FC) and 23/17 ± 3 °C	Regular irrigation (100% FC) and 23/17 ± 3 °C	Regular irrigation (100% FC) and 23/17 ± 3 °C without SeNPs application)
T2		Drought stress (60% FC) and 23/17 ± 3 °C	
T3		Regular irrigation (100% FC) and Heat stress (38 °C)	
T4		Regular irrigation (100% FC) and 23/17 ± 3 °C	Regular irrigation (100% FC) and 23/17 ± 3 °C with 10 mg·L^−1^ of SeNPs foliar application
T5		Drought stress (60% FC) and 23/17 ± 3 °C	
T6		Regular irrigation (100% FC) and Heat stress (38 °C)	

FC—Field Capacity, DAS—Days After Sown. Treatments: T1, control (regular irrigation, 100% Field Capacity (FC)) at (day/night) temperature of 23/17 ± 3 °C; T2, drought stress (60% FC); T3, heat stress (38 °C); T4, 10 mg·L^−1^ of SeNPs; T5, drought stress (60% FC) + 10 mg·L^−1^ of SeNPs; and T6, heat stress (38 °C) + 10 mg·L^−1^ of SeNPs.

## Data Availability

All data generated from this study are presented in the manuscript and Supplementary Materials.

## Supplementary Materials

The following supporting information can be downloaded at: https://www.mdpi.com/article/10.3390/nano13060998/s1, Figure S1: Characterization of SeNPs: (A) Transmission electron microscopy (TEM) microphotography of SeNPs; (B) Fourier transform infrared (FTIR) spectrum showing different potential functional groups responsible to stabilize or cap SeNPs. Arrows indicate different SeNPs sizes; Table S1: Name, pedigree, selection history, and year of release of the eight wheat genotypes and lines under study; Table S2: List of primers for the four metal-tolerant, phyto-chelating, and Zn transporter genes and the housekeeping gene (reference gene) and their sequences used in qRT-PCR analysis; Table S3: Descriptive statistics for morphological traits of eight wheat genotypes grown under control, drought, and heat stress conditions without and with SeNPs foliar application; Table S4: Performance of eight wheat genotypes morphological traits grown under control, drought, and heat stress conditions without and with SeNPs foliar application; Table S5: Total chlorophyll, photosynthetic rate, stomatal conductance, transpiration rate, electrolyte leakage, and leaf water potential of eight wheat genotypes under drought and heat stresses without and with SeNPs foliar application; Table S6: Changes in malondialdehyde (MDA), hydrogen peroxide (H_2_O_2_), proline contents, and in vivo changes in antioxidant enzyme activity, superoxide dismutase (SOD), ascorbate peroxidase (APX), and catalase (CAT), of eight wheat genotypes under drought and heat stresses without and with SeNPs foliar application; Table S7: Analysis of variance for the morpho-physiological and biochemical parameters across the main factors: eight studied wheat genotypes, condition, and their interaction; Table S8: Correlation of morpho-physiological traits of eight wheat genotypes under drought conditions.; Table S9: Correlation of morpho-physiological traits of eight wheat genotypes under SeNPs + drought conditions; Table S10: Correlation of morpho-physiological traits of eight wheat genotypes under heat conditions; Table S11: Correlation of morpho-physiological traits of eight wheat genotypes under SeNPs + heat conditions.
