# Supplementary material for: Conferring of Drought and Heat Stress Tolerance in Wheat (Triticum aestivum L.) Genotypes and Their Response to Selenium Nanoparticles Application
Source: Nanomaterials (Basel). 2023 Mar 9;13(6):998. doi: 10.3390/nano13060998 (PMC10051906; doi:10.3390/nano13060998)
Supplement: Supplementary file 1 [file nanomaterials-13-00998-s001.zip › nanomaterials-2222517-supplementary.pdf]

nanomaterials

MDPI

Supplementary Materials

Conferring of Drought and Heat Stress Tolerance in Wheat (*Triticum aestivum* L.) Genotypes and Their Response to Selenium Nanoparticles Application

Ahmad A. Omar [1,2,*,†], Yasmin M. Heikal [3], Ehab M. Zayed [4,†], Sahar A. M. Shamseldin [5], Yossry E. Salama [6], Khaled E. Amer [6], Mostafa M. Basuoni [7], Sawsan Abd Ellatif [8] and Azza H. Mohamed [2,9,*]

[1] Biochemistry Department, Faculty of Agriculture, Zagazig University, Zagazig 44519, Egypt
[2] Citrus Research and Education Center, University of Florida, IFAS, Lake Alfred, FL 33850, USA
[3] Botany Department, Faculty of Science, Mansoura University, Mansoura 35516, Egypt
[4] Cell Study Research Department, Field Crops Research Institute, Agricultural Research Center, Giza 12619, Egypt
[5] Botany Department, Women's College for Arts, Science and Education, Ain Shams University, Cairo 11566, Egypt
[6] Crop Science Department, Faculty of Agriculture, Damanhour University, Damanhour 22516, Egypt
[7] Botany and Microbiology Department, Faculty of Science (Boys), Al-Azhar University, Cairo 11884, Egypt
[8] Bioprocess Development Department, Genetic Engineering and Biotechnology Research Institute (GEBRI), City for Scientific Research and Technology Applications, New Borg El-Arab 21934, Egypt
[9] Agricultural Chemistry Department, Faculty of Agriculture, Mansoura University, Mansoura 33516, Egypt
* Correspondence: omar71@ufl.edu or aaelhanafi@agri.zu.edu.eg (A.A.O.); azza@ufl.edu or azza71@mans.edu.eg (A.H.M.); Tel.: +1-863-521-4569 (A.A.O.); +1-863-521-4886 (A.H.M.); Fax: +1-863-956-4631 (A.A.O. & A.H.M.)
† These authors contributed equally to this work.

Table S1. Name, pedigree, selection history, and year of release of the eight wheat genotypes and lines under study.

Genotypes	Pedigree	Selection History	Year of Release
Giza-168	MRL/BUC // SERI	CM93046-8M-0Y-0M-2Y-0B-0SH-0EGY	1999
Giza-171	SAKHA 93 / GEMMIZA 9	S.6-1GZ-4GZ-1GZ-2GZ-0EGY	2013
Misr-1	OASIS / SKAUZ // 4*BCN /3/ 2*PASTOR	CMSS00Y01881T-050M-030Y-030M-030WGY-33M-0Y-0EGY	2014
Misr-3	ATTILA*2/PBW65*2/KACHU	CMSS06Y00582T-099TOPM-099Y-099ZTM-099Y-099M-10WGY-0B-0EGY	2019
Shandweel-1	SITE / MO /4/ NAC / TH.AC // 3*PVN /3/ MIRLO / BUC	CMSS93B00567S-72Y-010M-010Y-010M-3Y-0M-0HTY-0SH-0EGY	2011
Sids-1	HD 2172 / Pavon "S" // 1158.57 / Maya 74 "S"	S 46-4SD-2SD-1SD-0SD-0EGY	1994
Sids-12	BUC // 7C / ALD /5/ MAYA74 / ON // 1160.147 /3/ BB / GLL /4/ CHAT"S" /6/ MAYA / VUL // CMH74A.630 / 4*SX	SD7096-4SD-1SD-1SD-0SD-0EGY	2009
Sids-14	BOW "S" / VEE"S" // BOW"S" / TSI/3/ BANI SEWEF 1	SD293-1SD-2SD-4SD-0SD-0EGY	2017

Table S2. List of primers for the four metal-tolerant, phyto-chelating, and Zn transporter genes and the housekeeping gene (reference gene) and their sequences used in qRT-PCR analysis.

Gene Name	Sequence (5′……………..3′)
Aquaporin (*PIP1*)	F: CTACATGATTGCGCAGTGCC<br>R: GCCGAACTGAACTGTCGAGA
Late embryogenesis abundant proteins (*LEA-1*)	F: CAGCGAAGTTTGGATGGAATG<br>R: ACCTGTCGCCAATCAGAAGAT
Heat shock protein 70 (*HSP 70*)	F: CAAGGGTGAGGACAAGCAGT<br>R: CCTCTGGGAATCATTGAAGTAA
Heat shock protein 90 (*HSP 90*)	F: CAAGTCGGACCTCGTCAACA<br>R: GAAACCGACACCAAACTGCC
β-Actin	F: GTGCCCATTTACGAAGGATA<br>R: GAAGACTCCATGCCGATCAT

Table S3. Descriptive statistics for morphological traits of eight wheat genotypes grown under control, drought, and heat stress conditions without and with SeNPs foliar application.

Traits	Treatments	Minimum	Maximum	Mean	SD
**PH (cm)**	T1	35.84	49.15	42.98	4.75
	T2	25.37	44.97	34.49	7.64
	T3	25.80	46.18	35.23	5.89
	T4	39.51	51.36	45.58	4.09
	T5	26.82	51.51	37.35	8.21
	T6	31.61	47.31	40.12	5.58
**SFW(g)**	T1	3.80	4.86	4.39	0.37
	T2	3.29	3.98	3.58	0.22
	T3	3.53	4.16	3.77	0.21
	T4	4.20	5.15	4.69	0.35
	T5	3.60	4.50	3.94	0.33
	T6	3.67	4.65	4.20	0.34
**SDW(g)**	T1	1.04	1.77	1.37	0.22
	T2	0.50	1.94	0.91	0.44
	T3	0.62	1.07	0.93	0.15
	T4	1.12	1.97	1.47	0.26
	T5	0.72	1.30	1.08	0.19
	T6	0.87	1.39	1.18	0.17
**RFW(g)**	T1	1.24	4.37	2.14	1.00
	T2	0.72	3.41	1.54	0.84
	T3	0.73	3.50	1.64	0.87
	T4	1.36	2.70	2.02	0.47
	T5	0.84	3.94	1.86	0.95
	T6	1.12	4.21	1.99	0.99
**RDW(g)**	T1	0.42	1.12	0.75	0.22
	T2	0.11	0.56	0.38	0.15
	T3	0.20	0.70	0.46	0.16
	T4	0.56	1.23	0.84	0.24
	T5	0.27	0.86	0.56	0.19
	T6	0.33	1.02	0.64	0.22

Plant height (PH), shoot fresh weight (SFW), shoot dry weight (SDW), root fresh weight (RFW), and root dry weight (RDW). Treatments: T1 = control (regular irrigation, 100% FC), T2 = drought stress (60% FC), T3 = heat stress (38°C), T4 = 10 mg.L^{-1} of SeNPs, T5 = drought stress + 10 mg.L^{-1} of SeNPs, and T6 = heat stress + 10 mg.L^{-1} of SeNPs.

Table S4. Performance of eight wheat genotypes morphological traits grown under control, drought, and heat stress conditions without and with SeNPs foliar application.

Genotypes	Treatments	PH	SFW	SDW	RFW	RDW
Giza-168	T1	35.84 ± 0.02b	4.09 ± 0.01b	1.77 ± 0.01b	1.24 ± 0.01b	0.94 ± 0.01b
	T2	25.84 ± 0.01f	3.41 ± 0.01f	0.90 ± 0.01f	0.72 ± 0.02f	0.55 ± 0.01f
	T3	26.48 ± 0.01e	3.53 ± 0.01e	1.03 ± 0.02e	0.79 ± 0.01e	0.63 ± 0.01e
	T4	40.15 ± 0.01a	5.15 ± 0.01a	1.97 ± 0.01a	1.54 ± 0.01a	1.11 ± 0.01a
	T5	27.63 ± 0.02d	3.64 ± 0.01d	1.18 ± 0.01d	0.84 ± 0.01d	0.73 ± 0.01d
	T6	31.61 ± 0.02c	3.97 ± 0.01c	1.34 ± 0.01c	1.12 ± 0.01c	0.82 ± 0.01c
Giza-171	T1	37.69 ± 0.01b	4.86 ± 0.02b	1.33 ± 0.03d	1.29 ± 0.01f	1.12 ± 0.01b
	T2	25.37 ± 0.01f	3.49 ± 0.02f	1.94 ± 0.01b	0.95 ± 0.01f	0.56 ± 0.01f
	T3	25.80 ± 0.01e	3.57 ± 0.01e	1.07 ± 0.01f	0.73 ± 0.59f	0.70 ± 0.01e
	T4	39.51 ± 0.01a	5.04 ± 0.01a	1.50 ± 0.01c	1.36 ± 0.02f	1.23 ± 0.02a
	T5	26.82 ± 0.01d	3.63 ± 0.01d	1.10 ± 0.01f	1.15 ± 0.01f	0.86 ± 0.01d
	T6	33.40 ± 0.02c	4.64 ± 0.02c	1.22 ± 0.01e	1.21 ± 0.01f	1.02 ± 0.01c
Misr-1	T1	43.16 ± 0.01c	4.08 ± 0.02b	1.30 ± 0.01b	1.96 ± 0.01b	0.76 ± 0.01c
	T2	44.97 ± 0.01b	3.63 ± 0.01f	0.74 ± 0.01f	1.41 ± 0.02f	0.39 ± 0.01f
	T3	37.74 ± 0.01e	3.75 ± 0.01e	0.98 ± 0.01e	1.47 ± 0.02e	0.41 ± 0.01f
	T4	46.45 ± 0.01a	4.20 ± 0.01a	1.33 ± 0.02a	2.05 ± 0.01a	0.81 ± 0.01b
	T5	37.62 ± 0.02f	3.87 ± 0.03d	1.07 ± 0.01d	1.60 ± 0.01d	0.54 ± 0.01e
	T6	40.49 ± 0.01d	4.02 ± 0.01c	1.15 ± 0.01c	1.70 ± 0.01c	0.61 ± 0.01d
Misr-3	T1	45.93 ± 0.01b	4.50 ± 0.02b	1.54 ± 0.01b	2.41 ± 0.01b	0.80 ± 0.01b
	T2	34.45 ± 0.01f	3.29 ± 0.01f	0.74 ± 0.01f	1.60 ± 0.01f	0.38 ± 0.01f
	T3	36.67 ± 0.02e	3.71 ± 0.02e	0.97 ± 0.02e	1.75 ± 0.01e	0.50 ± 0.01e
	T4	47.22 ± 0.01a	4.62 ± 0.01a	1.65 ± 0.01a	2.70 ± 0.01a	0.82 ± 0.01b
	T5	39.39 ± 0.01d	3.82 ± 0.02d	1.30 ± 0.01d	2.04 ± 0.01d	0.65 ± 0.01d
	T6	41.69 ± 0.01c	4.19 ± 0.01c	1.39 ± 0.01c	2.19 ± 0.02c	0.71 ± 0.01c
Shandaweel-1	T1	39.82 ± 0.02b	3.80 ± 0.01c	1.42 ± 0.01b	1.50 ± 0.01b	0.42 ± 0.02b
	T2	29.60 ± 0.01f	3.45 ± 0.01f	0.95 ± 0.01f	0.96 ± 0.01f	0.11 ± 0.01f
	T3	32.94 ± 0.01e	3.82 ± 0.01c	1.06 ± 0.01e	1.18 ± 0.01e	0.20 ± 0.01e
	T4	44.12 ± 0.02a	4.30 ± 0.01b	1.55 ± 0.01a	1.62 ± 0.03a	0.57 ± 0.01a
	T5	34.61 ± 0.01d	3.60 ± 0.01e	1.20 ± 0.01d	1.32 ± 0.01d	0.27 ± 0.01d
	T6	38.42 ± 0.01c	3.67 ± 0.01d	1.28 ± 0.01c	1.37 ± 0.01c	0.33 ± 0.01c
Sids-1	T1	47.15 ± 0.01c	4.62 ± 0.01c	1.04 ± 0.01b	2.17 ± 0.01c	0.76 ± 0.01b
	T2	43.81 ± 0.01f	3.98 ± 0.01f	0.50 ± 0.01f	1.53 ± 0.01f	0.42 ± 0.01f
	T3	46.18 ± 0.01e	3.96 ± 0.01f	0.62 ± 0.01e	1.87 ± 0.01e	0.51 ± 0.02e
	T4	48.59 ± 0.08b	4.74 ± 0.01b	1.12 ± 0.01a	2.20 ± 0.01b	0.87 ± 0.01a
	T5	51.51 ± 0.01a	4.20 ± 0.01e	0.72 ± 0.01d	2.07 ± 0.01d	0.61 ± 0.01d
	T6	46.52 ± 0.01d	4.37 ± 0.01d	0.87 ± 0.01c	2.09 ± 0.01d	0.67 ± 0.01c
Sids-12	T1	49.15 ± 0.01b	4.76 ± 0.01dd	1.19 ± 0.01b	4.37 ± 0.01a	0.70 ± 0.01b
	T2	39.50 ± 0.01e	3.78 ± 0.01f	0.72 ± 0.02f	3.41 ± 0.01e	0.30 ± 0.01f
	T3	31.08 ± 0.01f	4.16 ± 0.01fb	0.88 ± 0.01e	3.50 ± 0.01d	0.37 ± 0.01e
	T4	51.36 ± 0.01a	4.98 ± 0.01c	1.25 ± 0.01a	2.45 ± 0.01f	0.74 ± 0.01a
	T5	44.66 ± 0.01d	4.50 ± 0.34ec	0.98 ± 0.01d	3.94 ± 0.01c	0.44 ± 0.01d
	T6	47.31 ± 0.01c	4.65 ± 0.02dd	1.06 ± 0.01c	4.21 ± 0.01b	0.52 ± 0.01c
Sids-14	T1	45.12 ± 0.01b	4.37 ± 0.02b	1.33 ± 0.01b	2.16 ± 0.01b	0.52 ± 0.01b
	T2	32.35 ± 0.01f	3.59 ± 0.02f	0.76 ± 0.01f	1.74 ± 0.01f	0.29 ± 0.01f
	T3	36.81 ± 0.01d	3.65 ± 0.01e	0.85 ± 0.01e	1.80 ± 0.01e	0.34 ± 0.01e
	T4	47.20 ± 0.01a	4.49 ± 0.01a	1.40 ± 0.01a	2.20 ± 0.01a	0.56 ± 0.01a
	T5	36.54 ± 0.01e	3.89 ± 0.02d	0.98 ± 0.01d	1.89 ± 0.01d	0.38 ± 0.01d
	T6	41.53 ± 0.01c	4.12 ± 0.01c	1.12 ± 0.01c	2.01 ± 0.02c	0.44 ± 0.01c

Plant height (PH), shoot fresh weight (SFW), shoot dry weight (SDW), root fresh weight (RFW), and root dry weight (RDW). Data presented as means ± standard deviation (SD) of three replications (n = 3). Different letters indicate statistically significant differences among treatments according to (Tukey's HSD post hoc test) at $P \leq 0.05$. Treatments: T1 = control (regular irrigation, 100% FC), T2 = drought stress (60% FC), T3 = heat stress (38°C), T4 = 10 mg.L^{-1} of SeNPs, T5 = drought stress + 10 mg.L^{-1} of SeNPs, and T6 = heat stress + 10 mg.L^{-1} of SeNPs.

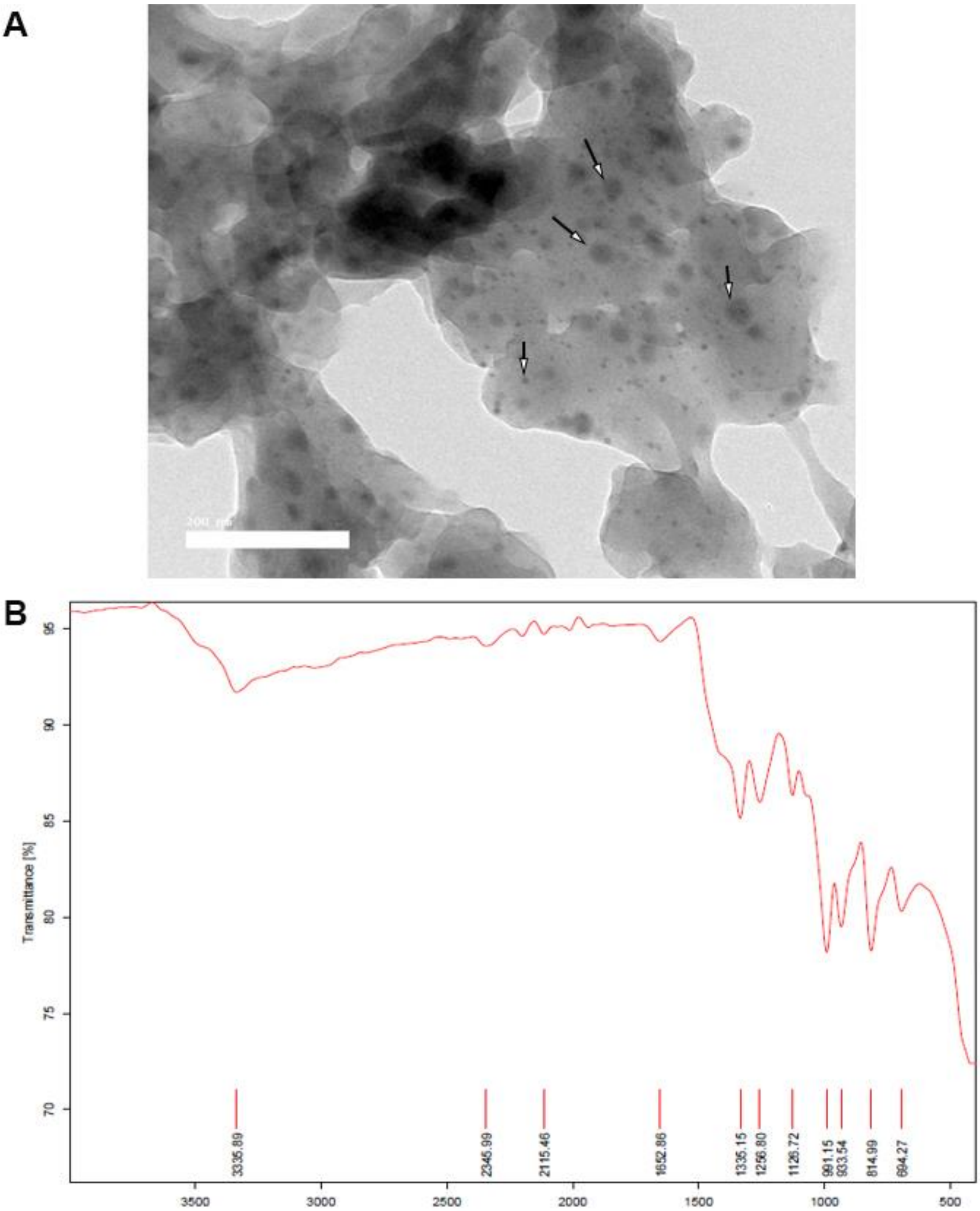

Figure S1. Characterization of SeNPs: (**A**) Transmission electron microscopy (TEM) microphotography of SeNPs; (**B**) Fourier transform infrared (FTIR) spectrum showing different potential functional groups responsible to stabilize or cap SeNPs. Arrows indicate different SeNPs sizes.

Table S5. Total chlorophyll, photosynthetic rate, stomatal conductance, transpiration rate, electrolyte leakage, and leaf water potential of eight wheat genotypes under drought and heat stresses without and with SeNPs foliar application.

Genotypes	Treatments	Giza-168	Giza-171	Misr-1	Misr-3	Shandaweel-1	Sids-1	Sids-12	Sids-14
**Total chlorophyll (CHLO)** (mg/g. FW)	T1	3.08 ± 0.01b	2.95 ± 0.01b	3.51 ± 0.01b	3.61 ± 0.01b	3.19 ± 0.01b	2.63 ± 0.01e	2.72 ± 0.01e	3.39 ± 0.01c
	T2	1.64 ± 0.01f	1.56 ± 0.02f	2.29 ± 0.01f	2.18 ± 0.01f	1.81 ± 0.01f	2.30 ± 0.01f	2.45 ± 0.01f	1.93 ± 0.01f
	T3	1.95 ± 0.01e	1.84 ± 0.01e	2.40 ± 0.01e	2.55 ± 0.01e	2.11 ± 0.01e	2.70 ± 0.01d	2.90 ± 0.01d	2.30 ± 0.01e
	T4	3.15 ± 0.01a	3.05 ± 0.02a	3.61 ± 0.01a	3.76 ± 0.01a	3.37 ± 0.01a	3.95 ± 0.01a	4.12 ± 0.02a	3.53 ± 0.01b
	T5	2.65 ± 0.01d	2.43 ± 0.02d	3.07 ± 0.01d	3.13 ± 0.02d	2.85 ± 0.01d	3.20 ± 0.01c	3.35 ± 0.01b	2.96 ± 0.01d
	T6	2.74 ± 0.01c	2.55 ± 0.01c	3.22 ± 0.01c	3.34 ± 0.01c	2.94 ± 0.01c	3.45 ± 0.01b	3.20 ± 0.01c	2.95 ± 0.01d
**Photosynthetic rate (PN)** (μmol (CO_2) m^{-2} s^{-1})	T1	6.17 ± 0.01b	6.01 ± 0.02b	6.51 ± 0.01c	6.63 ± 0.01b	6.34 ± 0.01b	6.71 ± 0.01b	6.82 ± 0.02c	6.49 ± 0.01b
	T2	4.87 ± 0.01f	4.80 ± 0.01f	5.21 ± 0.01f	5.31 ± 0.01f	5.06 ± 0.01f	5.42 ± 0.02f	5.52 ± 0.02f	5.12 ± 0.01e
	T3	5.02 ± 0.01e	5.02 ± 0.01e	5.43 ± 0.01e	5.54 ± 0.01e	5.20 ± 0.01e	5.63 ± 0.01e	5.72 ± 0.01e	5.32 ± 0.01d
	T4	6.27 ± 0.01a	6.12 ± 0.01a	6.81 ± 0.07b	6.70 ± 0.01a	6.40 ± 0.01a	6.84 ± 0.01a	6.98 ± 0.01b	6.53 ± 0.01a
	T5	5.95 ± 0.01d	5.72 ± 0.01d	6.40 ± 0.01d	6.46 ± 0.02d	6.10 ± 0.01d	6.50 ± 0.01d	6.71 ± 0.01d	6.18 ± 0.01c
	T6	6.03 ± 0.01c	6.03 ± 0.01c	6.42 ± 0.01d	6.52 ± 0.01c	6.15 ± 0.01c	6.63 ± 0.01c	6.73 ± 0.02d	6.25 ± 0.01f
**Stomatal conductance (SC)** (mmol H_2O $m^{-2}s^{-2}$)	T1	27.34 ± 0.01b	26.91 ± 0.01b	27.81 ± 0.01b	28.09 ± 0.02b	27.61 ± 0.01b	28.12 ± 0.01b	28.30 ± 0.01b	27.72 ± 0.02b
	T2	17.74 ± 0.01d	17.65 ± 0.01d	11.26 ± 0.02f	18.60 ± 0.19d	17.96 ± 0.02d	18.61 ± 0.01d	12.71 ± 0.02f	18.12 ± 0.02d
	T3	16.81 ± 0.01e	16.51 ± 0.01e	17.19 ± 0.01d	17.33 ± 0.01e	16.86 ± 0.02e	17.51 ± 0.02e	18.01 ± 0.02d	17.04 ± 0.01e
	T4	28.80 ± 0.01a	28.61 ± 0.01a	29.45 ± 0.01a	29.61 ± 0.01a	29.08 ± 0.02a	29.01 ± 0.02a	31.61 ± 0.01a	29.30 ± 0.02a
	T5	13.71 ± 0.01f	13.59 ± 0.01f	14.21 ± 0.01e	14.30 ± 0.01f	13.94 ± 0.01f	19.91 ± 0.01f	14.51 ± 0.01e	14.12 ± 0.02f
	T6	18.92 ± 0.02c	18.82 ± 0.02c	19.60 ± 0.02c	19.76 ± 0.01c	13.94 ± 0.01c	19.91 ± 0.01c	20.30 ± 0.02c	19.33 ± 0.01c
**Transpiration rate (TR)** (mmol H_2O $m^{-2}s^{-2}$)	T1	0.73 ± 0.01c	0.77 ± 0.00c	0.80 ± 0.01c	0.81 ± 0.01c	0.75 ± 0.02d	0.80 ± 0.01c	0.81 ± 0.01d	0.76 ± 0.01c
	T2	0.60 ± 0.02d	0.61 ± 0.01ec	0.70 ± 0.01d	0.71 ± 0.01d	0.65 ± 0.01e	0.75 ± 0.01d	0.79 ± 0.02d	0.67 ± 0.01d
	T3	0.55 ± 0.01e	0.59 ± 0.01e	0.68 ± 0.02d	0.7 ± 0.01d	0.62 ± 0.01e	0.73 ± 0.01d	0.74 ± 0.01e	0.66 ± 0.01d
	T4	0.75 ± 0.01c	0.80 ± 0.01b	0.84 ± 0.01d	0.82 ± 0.01c	0.78 ± 0.02d	0.88 ± 0.01b	0.90 ± 0.01c	0.80 ± 0.01b
	T5	0.47 ± 0.01f	0.51 ± 0.01f	0.51 ± 0.01f	0.54 ± 0.01f	0.52 ± 0.01f	0.52 ± 0.01f	0.68 ± 0.02f	0.49 ± 0.01f
	T6	0.59 ± 0.01d	0.62 ± 0.01d	0.61 ± 0.01e	0.65 ± 0.01e	0.62 ± 0.01e	0.69 ± 0.01e	0.70 ± 0.02f	0.62 ± 0.01e
**Electrolyte Leakage (EL)** (%)	T1	37.75 ± 0.01e	37.51 ± 0.01d	38.44 ± 0.01e	39.19 ± 0.01e	37.95 ± 0.01e	39.41 ± 0.01f	39.61 ± 0.01e	39.24 ± 0.02e
	T2	74.51 ± 0.01a	74.72 ± 0.02a	73.74 ± 0.01a	73.54 ± 0.02a	74.07 ± 0.01a	73.35 ± 0.01a	73.15 ± 0.01a	73.91 ± 0.01a
	T3	57.13 ± 0.01d	37.30 ± 0.01f	56.72 ± 0.02d	65.60 ± 0.01c	56.91 ± 0.01d	65.41 ± 0.01b	65.22 ± 0.01b	56.81 ± 0.01d
	T4	23.90 ± 0.01f	33.62 ± 0.02e	34.80 ± 0.02f	35.04 ± 0.02f	34.55 ± 0.02f	63.43 ± 0.02c	39.51 ± 0.01f	34.63 ± 0.02f
	T5	57.91 ± 0.01c	58.16 ± 0.01c	57.69 ± 0.03c	71.19 ± 0.01b	57.84 ± 0.01c	57.40 ± 0.01e	57.61 ± 0.01d	57.74 ± 0.01c
	T6	63.12 ± 0.02b	63.30 ± 0.01b	62.65 ± 0.01b	62.45 ± 0.01d	62.77 ± 0.02b	62.31 ± 0.01d	62.14 ± 0.01c	62.80 ± 0.01b

**Leaf water potential (LWP)** (MPa)	T1	0.37 ± 0.01e	0.37 ± 0.01f	0.33 ± 0.02e	0.31 ± 0.01e	0.35 ± 0.01f	0.32 ± 0.02e	0.32 ± 0.01e	0.21 ± 0.02f
	T2	1.02 ± 0.01d	1.05 ± 0.01ec	1.12 ± 0.01b	0.15 ± 0.11f	1.15 ± 0.01ec	1.05 ± 0.03c	1.03 ± 0.02b	1.11 ± 0.02c
	T3	1.00 ± 0.01d	1.03 ± 0.02e	1.04 ± 0.01d	1.03 ± 0.01d	1.11 ± 0.01e	0.96 ± 0.02d	0.92 ± 0.02d	1.05 ± 0.02d
	T4	0.34 ± 0.01f	0.35 ± 0.01f	0.28 ± 0.01f	0.25 ± 0.01fb	0.32 ± 0.02f	0.26 ± 0.01f	0.25 ± 0.01f	0.28 ± 0.01e
	T5	1.05 ± 0.01c	1.08 ± 0.01d	1.18 ± 0.01a	1.14 ± 0.01d	1.18 ± 0.06d	1.12 ± 0.01b	1.11 ± 0.02a	1.20 ± 0.01b
	T6	1.05 ± 0.01c	1.12 ± 0.02c	1.08 ± 0.01c	1.06 ± 0.02d	1.14 ± 0.01ec	0.98 ± 0.01d	0.97 ± 0.01c	1.18 ± 0.01b

Data presented as means ± standard deviation (SD) of three replications (*n* = 3). Different letters indicate statistically significant differences among treatments according to (Tukey's HSD post hoc test) at P ≤ 0.05. Treatments: T1 = control (regular irrigation, 100% FC), T2 = drought stress (60% FC), T3 = heat stress (38°C), T4 = 10 mg.L^{-1} of SeNPs, T5 = drought stress + 10 mg.L^{-1} of SeNPs, and T6 = heat stress + 10 mg.L^{-1} of SeNPs.

Table S6. Changes in malondialdehyde (MDA), hydrogen peroxide (H_2O_2), proline contents, and in vivo changes in antioxidant enzyme activity, superoxide dismutase (SOD), ascorbate peroxidase (APX), and catalase (CAT), of eight wheat genotypes under drought and heat stresses without and with SeNPs foliar application.

Genotypes	Treatments	MDA (µmol.g^{-1} FW)	H_2O_2 (µmol.g^{-1} FW)	Proline (µmol g^{-1} FW)	SOD (U.g^{-1} FW. min^{-1})	APX (µmol AsA g^{-1}. FW. min^{-1})	CAT (µmol H_2O_2 g^{-1}. FW. min^{-1})
Giza-168	T1	2.54 ± 0.01f	2.05 ± 0.01e	1.80 ± 0.01e	145.67 ± 0.06f	71.17 ± 0.06f	26.17 ± 0.06f
	T2	9.12 ± 0.01d	3.41 ± 0.01b	2.63 ± 0.01d	361.43 ± 0.06b	129.57 ± 0.06b	62.13 ± 0.06b
	T3	9.26 ± 0.01b	3.28 ± 0.01c	2.71 ± 0.01c	358.63 ± 1.76c	128.57 ± 0.06d	53.13 ± 0.06d
	T4	2.65 ± 0.01e	1.78 ± 0.01f	1.24 ± 0.58f	152.30 ± 0.00e	74.17 ± 0.06e	26.77 ± 0.06e
	T5	9.22 ± 0.01c	3.15 ± 0.01d	2.74 ± 0.01b	331.40 ± 0.10d	131.20 ± 0.10a	63.17 ± 0.06a
	T6	9.35 ± 0.02a	4.00 ± 0.02a	2.86 ± 0.02a	369.77 ± 0.06a	129.73 ± 0.06c	54.57 ± 0.06c
Giza-171	T1	2.60 ± 0.01f	2.11 ± 0.01d	1.85 ± 0.01b	148.80 ± 0.10f	73.43 ± 0.06e	26.83 ± 0.06e
	T2	9.31 ± 0.02b	3.55 ± 0.01a	2.69 ± 0.01a	362.73 ± 0.06c	136.33 ± 1.79b	63.40 ± 0.10b
	T3	9.29 ± 0.01d	3.42 ± 0.01b	2.69 ± 0.01a	360.03 ± 0.06d	131.13 ± 0.06d	55.50 ± 0.10c
	T4	2.71 ± 0.01e	1.80 ± 0.01e	1.28 ± 0.59b	156.23 ± 0.06e	72.50 ± 0.10f	28.10 ± 0.10d
	T5	9.42 ± 0.01a	0.25 ± 0.01f	2.76 ± 0.01a	378.50 ± 0.10a	137.57 ± 0.06a	64.70 ± 0.10a
	T6	9.37 ± 0.01c	3.12 ± 0.01c	2.91 ± 0.01a	374.33 ± 0.06b	133.73 ± 0.06c	55.50 ± 0.10c
Misr-1	T1	2.87 ± 0.01f	1.68 ± 0.01e	1.63 ± 0.01f	178.50 ± 0.61f	57.77 ± 0.06f	27.13 ± 0.06e
	T2	7.07 ± 0.01c	3.20 ± 0.02a	2.28 ± 0.02c	370.77 ± 0.06c	123.60 ± 0.10b	51.23 ± 0.06b
	T3	6.71 ± 0.01d	3.12 ± 0.02b	2.30 ± 0.01b	363.13 ± 0.06d	115.40 ± 0.10d	51.23 ± 0.06b
	T4	2.91 ± 0.01e	1.52 ± 0.02f	1.80 ± 0.01e	184.17 ± 0.06e	58.17 ± 0.06e	28.87 ± 0.06d
	T5	7.34 ± 0.02b	3.08 ± 0.01c	2.17 ± 0.01d	389.13 ± 0.06a	125.20 ± 0.10a	55.17 ± 0.06a
	T6	7.86 ± 0.02a	2.81 ± 0.01d	2.33 ± 0.02a	371.40 ± 0.10b	116.07 ± 0.06c	50.60 ± 0.10b
Misr-3	T1	2.74 ± 0.02e	1.65 ± 0.01e	1.61 ± 0.01d	171.20 ± 0.10f	57.33 ± 0.06f	27.07 ± 0.06f
	T2	6.97 ± 0.01b	3.19 ± 0.01a	2.21 ± 0.01b	365.13 ± 0.06c	119.43 ± 0.06b	50.87 ± 0.06c

	T3	6.50 ± 0.01c	3.06 ± 0.02b	2.19 ± 0.01b	360.47 ± 0.06d	112.53 ± 0.06d	43.30 ± 0.10d
	T4	2.82 ± 0.02d	1.53 ± 0.01f	1.75 ± 0.01c	180.10 ± 0.10e	58.13 ± 0.06e	28.17 ± 0.06e
	T5	7.12 ± 0.01a	3.02 ± 0.01c	2.15 ± 0.01d	386.07 ± 0.06a	124.87 ± 0.06a	51.17 ± 0.06a
	T6	6.51 ± 0.02c	2.76 ± 0.02d	2.30 ± 0.01a	369.37 ± 0.06b	115.63 ± 0.06c	43.53 ± 0.06c
Shandaweel-1	T1	4.05 ± 0.01e	1.90 ± 0.01e	1.31 ± 0.01f	187.17 ± 0.06e	69.50 ± 0.10e	28.67 ± 0.06e
	T2	8.05 ± 0.01c	3.31 ± 0.01a	2.15 ± 0.01d	373.40 ± 0.10c	137.13 ± 0.06b	50.77 ± 0.06b
	T3	7.62 ± 0.01d	3.19 ± 0.01b	2.19 ± 0.01c	371.53 ± 0.06d	125.13 ± 0.06d	49.27 ± 0.06c
	T4	3.19 ± 0.01f	1.63 ± 0.02f	1.42 ± 0.01e	178.17 ± 11.55e	70.13 ± 0.06e	29.83 ± 0.06d
	T5	8.41 ± 0.01b	3.11 ± 0.01c	2.25 ± 0.01b	398.03 ± 0.06a	136.07 ± 0.06c	53.17 ± 0.06a
	T6	8.8 ± 0.01a	2.90 ± 0.02d	2.30 ± 0.01a	378.37 ± 0.06b	173.43 ± 0.06a	50.87 ± 0.06b
Sids-1	T1	2.7 ± 0.01e	1.61 ± 0.01e	1.35 ± 0.01f	186.33 ± 0.06f	66.17 ± 0.06e	28.50 ± 0.10f
	T2	6.85 ± 0.01b	3.16 ± 0.01a	2.05 ± 0.01c	371.70 ± 0.10b	121.47 ± 0.06b	52.40 ± 0.10b
	T3	6.32 ± 0.01d	3.06 ± 0.01b	2.14 ± 0.01b	369.47 ± 0.06c	114.53 ± 0.06c	42.63 ± 0.06d
	T4	2.71 ± 0.01e	1.49 ± 0.01f	1.49 ± 0.01e	192.90 ± 0.52e	65.07 ± 0.06f	29.13 ± 0.06e
	T5	6.93 ± 0.01a	2.91 ± 0.01c	1.95 ± 0.01d	405.13 ± 0.06a	121.77 ± 0.06a	54.20 ± 0.10a
	T6	6.51 ± 0.01c	2.72 ± 0.01d	2.15 ± 0.01a	338.33 ± 0.06d	113.87 ± 0.06d	45.63 ± 0.06c
Sids-12	T1	2.61 ± 0.02f	1.57 ± 0.01e	1.35 ± 0.01e	187.53 ± 0.06f	66.07 ± 0.06e	29.30 ± 0.61e
	T2	6.75 ± 0.01b	3.12 ± 0.01a	2.04 ± 0.01b	370.47 ± 0.06c	121.27 ± 0.06b	51.30 ± 0.10b
	T3	6.21 ± 0.01d	2.91 ± 0.01b	2.14 ± 0.02a	365.57 ± 0.06d	111.07 ± 0.06c	43.50 ± 0.10d
	T4	2.71 ± 0.01e	1.41 ± 0.01f	1.41 ± 0.01d	196.43 ± 0.06e	69.47 ± 0.06e	29.30 ± 0.10e
	T5	6.87 ± 0.01a	2.89 ± 0.01c	1.94 ± 0.01c	395.57 ± 0.06a	123.17 ± 0.06a	53.13 ± 0.06a
	T6	6.41 ± 0.01c	2.67 ± 0.01d	2.04 ± 0.02b	371.07 ± 0.06b	111.33 ± 0.06c	45.37 ± 0.21c
Sids-14	T1	2.85 ± 0.01f	0.77 ± 0.01f	1.41 ± 0.02e	175.23 ± 0.06f	67.07 ± 0.06e	28.67 ± 0.06f
	T2	7.18 ± 0.01c	3.24 ± 0.01a	2.11 ± 0.01d	381.43 ± 0.06b	123.57 ± 0.06b	52.27 ± 2.80b
	T3	6.92 ± 0.01d	3.15 ± 0.01b	2.16 ± 0.01c	374.17 ± 0.06d	115.40 ± 0.10c	49.20 ± 0.10d
	T4	2.91 ± 0.01e	1.57 ± 0.03e	1.21 ± 0.59f	179.17 ± 0.06e	68.23 ± 0.06e	29.90 ± 0.10e
	T5	7.53 ± 0.03b	3.11 ± 0.01c	2.20 ± 0.01b	396.47 ± 0.06a	125.20 ± 0.10a	57.13 ± 0.06a
	T6	7.99 ± 0.02a	2.86 ± 0.02d	2.27 ± 0.01a	376.13 ± 0.06c	115.13 ± 0.06c	50.80 ± 0.10c

Data presented as means ± standard deviation (SD) of three replications (n = 3). Different letters indicate statistically significant differences among treatments according to (Tukey's HSD post hoc test) at $P \leq 0.05$. Abbreviations: MDA—malondialdehyde, H_2O_2—hydrogen peroxide, SOD—superoxide dismutase, APX—ascorbate peroxidase, AsA—ascorbate, and CAT—catalase. Treatments: T1, control (regular irrigation, 100% Field Capacity (FC)) at (day/night) temperature of 23/17 ± 3°C; T2, drought stress (60% FC); T3, heat stress (38°C); T4, 10 $mg.L^{-1}$ of SeNPs; T5, drought stress (60% FC) + 10 $mg.L^{-1}$ of SeNPs; and T6, heat stress (38°C) + 10 $mg.L^{-1}$ of SeNPs.

Table S7. Analysis of variance for the morpho-physiological and biochemical parameters across the main factors: eight studied wheat genotypes, condition, and their interaction.

Parameters	Variable and Source of Variation	df	MS	F	P
**PH**	Intercept	1	220384.9	431.947	0
	Genotypes	7	583.912	21.274	0
	Condition	5	510.212	18.589	0
	Genotypes × Condition	35	27.447	1.12E+05	0
**SFW**	Intercept	1	2404.268	577.84	0
	Genotypes	7	0.927	6.076	0
	Condition	5	4.161	27.287	0
	Genotypes × Condition	35	0.152	58.383	0
**SDW**	Intercept	1	191.546	150.828	0
	Genotypes	7	0.653	6.199	0
	Condition	5	1.27	12.058	0
	Genotypes × Condition	35	0.105	924.787	0
**RFW**	Intercept	1	499.522	385.317	0
	Genotypes	7	12.401	48.112	0
	Condition	5	1.296	5.03	0.001
	Genotypes × Condition	35	0.258	35.411	0
**RDW**	Intercept	1	52.478	70.85	0
	Genotypes	7	0.672	88.347	0
	Condition	5	0.741	97.325	0
	Genotypes × Condition	35	0.008	75.063	0
**Total chlorophyll**	Intercept	1	1165.255	152.537	0
	Genotypes	7	1.34	8.891	0
	Condition	5	7.639	50.669	0
	Genotypes × Condition	35	0.151	1.47E+03	0
**Photosynthetic rate**	Intercept	1	5078.106	602.243	0
	Genotypes	7	2.632	2.536	0.032
	Condition	5	8.432	8.126	0
	Genotypes × Condition	35	1.038	4.71E+03	0
**Stomatal conductance**	Intercept	1	60743.76	59.663	0.001
	Genotypes	7	17.197	0.896	0.52
	Condition	5	1018.112	53.065	0
	Genotypes × Condition	35	19.186	2.22E+04	0
**Transpiration rate**	Intercept	1	67.596	258.079	0
	Genotypes	7	0.044	20.041	0
	Condition	5	0.262	120.458	0
	Genotypes × Condition	35	0.002	17.017	0
**Electrolyte leakage**	Intercept	1	425024.7	87.674	0
	Genotypes	7	328.582	1.317	0.272
	Condition	5	4847.786	19.431	0
	Genotypes × Condition	35	249.493	1.61E+06	0
**Leaf water potential**	Intercept	1	91.904	25.703	0.004
	Genotypes	7	0.092	1.367	0.25
	Condition	5	3.576	53.363	0

	Genotypes × Condition	35	0.067	510.512	0
**Malondialdehyde (MDA)**	Intercept	1	5305.059	35.323	0.002
	Genotypes	7	10.493	8.348	0
	Condition	5	150.188	119.48	0
	Genotypes × Condition	35	1.257	19.088	0
**Hydrogen peroxide (H2O2)**	Intercept	1	942.644	70.649	0
	Genotypes	7	0.592	0.817	0.58
	Condition	5	13.343	18.422	0
	Genotypes × Condition	35	0.724	5.58E+03	0
**Proline**	Intercept	1	600.985	136.777	0
	Genotypes	7	0.819	8.515	0
	Condition	5	4.394	45.688	0
	Genotypes × Condition	35	0.096	4.5	0
**Superoxide dismutase (SOD)**	Intercept	1	1.24E+07	59.732	0.001
	Genotypes	7	15478.93	0.999	0.449
	Condition	5	208291.3	13.439	0
	Genotypes × Condition	35	15498.67	5.42E+03	0
**Ascorbate peroxidase (APX)**	Intercept	1	1540371	73.024	0
	Genotypes	7	2161.71	2.557	0.031
	Condition	5	21094.05	24.948	0
	Genotypes × Condition	35	845.523	1.19E+04	0
**Catalase (CAT)**	Intercept	1	280026.2	72.787	0
	Genotypes	7	150.89	5.339	0
	Condition	5	3847.205	136.12	0
	Genotypes × Condition	35	28.263	158.857	0

Plant height (PH), shoot fresh weight (SFW), shoot dry weight (SDW), root fresh weight (RFW), and root dry weight (RDW). df—degree of freedom, MS—mean square, F—F statistics, P—probability level.

Table S8. Correlation of morpho-physiological traits of eight wheat genotypes under drought conditions.

	**PH**	**SFW**	**RFW**	**SDW**	**RDW**	**CHLO**	**PN**	**SC**	**TR**	**EL**	**LWP**	**MDA**	**H_2O_2**	**PRO**	**SOD**	**APX**	**CAT**
**PH**	1.00000	0.69562	0.50914	−0.66821	−0.19443	0.91843	0.82468	−0.59521	0.83952	−0.82643	0.01796	−0.84326	−0.82515	−0.67008	0.36228	−0.68777	−0.71293
**SFW**	0.69562	1.00000	0.47668	−0.38372	−0.04365	0.58339	0.59590	−0.19539	0.65266	−0.56510	0.49095	−0.49244	−0.49041	−0.51498	0.39713	−0.27655	−0.37167
**RFW**	0.50914	0.47668	1.00000	−0.38677	−0.30150	0.75144	0.78837	−0.20598	0.83453	−0.77453	−0.05833	−0.68140	−0.69091	−0.63081	0.31770	−0.50317	−0.60882
**SDW**	−0.66821	−0.38372	−0.38677	1.00000	0.41102	−0.72304	−0.74082	0.16528	−0.63540	0.77172	0.13605	0.78152	0.88306	0.75658	−0.45488	0.73557	0.73370
**RDW**	−0.19443	−0.04365	−0.30150	0.41102	1.00000	−0.30474	−0.39169	0.00880	−0.34290	0.43056	−0.10407	0.48859	0.49609	0.74344	−0.70229	0.78642	−0.01427
**CHLO**	0.91843	0.58339	0.75144	−0.72304	−0.30474	1.00000	0.96351	−0.44978	0.96433	−0.96420	−0.19217	−0.92929	−0.93735	−0.77675	0.32882	−0.77102	−0.82024
**PN**	0.82468	0.59590	0.78837	−0.74082	−0.39169	0.96351	1.00000	−0.20777	0.98522	−0.99715	−0.23347	−0.93010	−0.94825	−0.86060	0.37088	−0.78810	−0.79334
**SC**	−0.59521	−0.19539	−0.20598	0.16528	0.00880	−0.44978	−0.20777	1.00000	−0.27087	0.21954	−0.25592	0.29322	0.28308	0.05495	−0.06961	0.26438	0.22211
**TR**	0.83952	0.65266	0.83453	−0.63540	−0.34290	0.96433	0.98522	−0.27087	1.00000	−0.97488	−0.16426	−0.90263	−0.89995	−0.81810	0.35422	−0.73632	−0.75575
**EL**	−0.82643	−0.56510	−0.77453	0.77172	0.43056	−0.96420	−0.99715	0.21954	−0.97488	1.00000	0.24358	0.95127	0.96784	0.88249	−0.41236	0.82522	0.80462
**LWP**	0.01796	0.49095	−0.05833	0.13605	−0.10407	−0.19217	−0.23347	−0.25592	−0.16426	0.24358	1.00000	0.23712	0.21081	0.04938	0.35904	0.18391	0.43936
**MDA**	−0.84326	−0.49244	−0.68140	0.78152	0.48859	−0.92929	−0.93010	0.29322	−0.90263	0.95127	0.23712	1.00000	0.96903	0.90516	−0.58370	0.90047	0.82467
**H_2O_2**	−0.82515	−0.49041	−0.69091	0.88306	0.49609	−0.93735	−0.94825	0.28308	−0.89995	0.96784	0.21081	0.96903	1.00000	0.89960	−0.51105	0.88263	0.82389
**PRO**	−0.67008	−0.51498	−0.63081	0.75658	0.74344	−0.77675	−0.86060	0.05495	−0.81810	0.88249	0.04938	0.90516	0.89960	1.00000	−0.73903	0.92518	0.57609
**SOD**	0.36228	0.39713	0.31770	−0.45488	−0.70229	0.32882	0.37088	−0.06961	0.35422	−0.41236	0.35904	−0.58370	−0.51105	−0.73903	1.00000	−0.66295	−0.23354
**APX**	−0.68777	−0.27655	−0.50317	0.73557	0.78642	−0.77102	−0.78810	0.26438	−0.73632	0.82522	0.18391	0.90047	0.88263	0.92518	−0.66295	1.00000	0.54647
**CAT**	−0.71293	−0.37167	−0.60882	0.73370	−0.01427	−0.82024	−0.79334	0.22211	−0.75575	0.80462	0.43936	0.82467	0.82389	0.57609	−0.23354	0.54647	1.00000

Plant height (PH), shoot fresh weight (SFW), shoot dry weight (SDW), root fresh weight (RFW), root dry weight (RDW), total chlorophyll (CHLO), net photosynthetic rate (PN), stomatal conductance (SC), transpiration rate (TR), electrolyte leakage (EL), leaf water potential (LWP), malondialdehyde (MDA), hydrogen peroxide (H_2O_2), proline (PRO), superoxide dismutase (SOD), ascorbate peroxidase (APX), and catalase (CAT).

Table S9. Correlation of morpho-physiological traits of eight wheat genotypes under SeNPs + drought conditions.

	PH	SFW	RFW	SDW	RDW	CHLO	PN	SC	TR	EL	LWP	MDA	H_2O_2	PRO	SOD	APX	CAT
**PH**	1.00000	0.81510	0.68413	−0.66463	−0.34823	0.89346	0.87462	0.91792	0.49658	0.05594	0.27322	−0.89835	0.44099	−0.92281	0.52003	−0.74487	−0.81532
**SFW**	0.81510	1.00000	0.92320	−0.64424	−0.23260	0.82311	0.83661	0.83990	0.79627	−0.12613	−0.01878	−0.77085	0.25762	−0.76034	0.35258	−0.48117	−0.77461
**RFW**	0.68413	0.92320	1.00000	−0.35800	−0.35297	0.80389	0.81939	0.81461	0.93326	0.05054	0.08592	−0.74161	0.22398	−0.74691	0.44930	−0.57703	−0.64175
**SDW**	−0.66463	−0.64424	−0.35800	1.00000	0.08185	−0.38424	−0.34846	−0.42744	−0.16095	0.56315	−0.01057	0.42173	−0.02241	0.46055	-0.29075	0.05672	0.51110
**RDW**	−0.34823	−0.23260	−0.35297	0.08185	1.00000	−0.49708	−0.41055	−0.44458	−0.25735	0.20800	−0.75609	0.44430	−0.62911	0.61132	-0.39408	0.64296	0.19900
**CHLO**	0.89346	0.82311	0.80389	−0.38424	−0.49708	1.00000	0.99144	0.99296	0.63272	0.18736	0.37329	−0.96862	0.63948	−0.95511	0.44096	−0.85878	−0.87609
**PN**	0.87462	0.83661	0.81939	−0.34846	−0.41055	0.99144	1.00000	0.98519	0.67739	0.22053	0.28423	−0.95018	0.59375	−0.92216	0.40628	−0.83345	−0.86836
**SC**	0.91792	0.83990	0.81461	−0.42744	−0.44458	0.99296	0.98519	1.00000	0.63535	0.20917	0.37240	−0.98420	0.56040	−0.96231	0.50978	−0.84675	−0.88633
**TR**	0.49658	0.79627	0.93326	−0.16095	−0.25735	0.63272	0.67739	0.63535	1.00000	0.04881	−0.07988	−0.52679	0.04958	−0.56840	0.38056	−0.47549	−0.37502
**EL**	0.05594	−0.12613	0.05054	0.56315	0.20800	0.18736	0.22053	0.20917	0.04881	1.00000	0.06874	−0.24778	0.10273	−0.11097	0.10657	−0.40196	−0.17755
**LWP**	0.27322	−0.01878	0.08592	−0.01057	−0.75609	0.37329	0.28423	0.37240	−0.07988	0.06874	1.00000	−0.48114	0.41595	−0.54337	0.64613	−0.60701	−0.22533
**MDA**	−0.89835	−0.77085	−0.74161	0.42173	0.44430	−0.96862	−0.95018	−0.98420	−0.52679	−0.24778	−0.48114	1.00000	−0.55478	0.95834	−0.56680	0.84575	0.90183
**H_2O_2**	0.44099	0.25762	0.22398	−0.02241	−0.62911	0.63948	0.59375	0.56040	0.04958	0.10273	0.41595	−0.55478	1.00000	−0.55967	−0.14467	−0.62951	−0.57763
**PRO**	−0.92281	−0.76034	−0.74691	0.46055	0.61132	−0.95511	−0.92216	−0.96231	−0.56840	−0.11097	−0.54337	0.95834	−0.55967	1.00000	−0.64025	0.89765	0.78016
**SOD**	0.52003	0.35258	0.44930	−0.29075	−0.39408	0.44096	0.40628	0.50978	0.38056	0.10657	0.64613	−0.56680	−0.14467	−0.64025	1.00000	−0.57658	−0.23576
**APX**	−0.74487	−0.48117	−0.57703	0.05672	0.64296	−0.85878	−0.83345	−0.84675	−0.47549	−0.40196	−0.60701	0.84575	−0.62951	0.89765	−0.57658	1.00000	0.58749
**CAT**	−0.81532	−0.77461	−0.64175	0.51110	0.19900	−0.87609	−0.86836	−0.88633	−0.37502	−0.17755	−0.22533	0.90183	−0.57763	0.78016	−0.23576	0.58749	1.00000

Plant height (PH), shoot fresh weight (SFW), shoot dry weight (SDW), root fresh weight (RFW), root dry weight (RDW), total chlorophyll (CHLO), net photosynthetic rate (PN), stomatal conductance (SC), transpiration rate (TR), electrolyte leakage (EL), leaf water potential (LWP), malondialdehyde (MDA), hydrogen peroxide (H_2O_2), proline (PRO), superoxide dismutase (SOD), ascorbate peroxidase (APX), and catalase (CAT).

Table S10. Correlation of morpho-physiological traits of eight wheat genotypes under heat conditions.

	PH	SFW	RFW	SDW	RDW	CHLO	PN	SC	TR	EL	LWP	MDA	H_2O_2	PRO	SOD	APX	CAT
**PH**	1.00000	0.41020	0.29064	−0.81010	−0.32359	0.62131	0.69822	0.47283	0.70295	0.56905	−0.13721	−0.77552	−0.54749	−0.74153	0.52001	−0.76743	0.03430
**SFW**	0.41020	1.00000	0.86411	−0.54192	−0.47704	0.85210	0.80755	0.87771	0.81136	0.49388	−0.56849	−0.73156	−0.82681	−0.69071	0.36611	−0.73134	−0.80738
**RFW**	0.29064	0.86411	1.00000	−0.50084	−0.41041	0.90000	0.84215	0.94077	0.83535	0.53686	−0.64756	−0.76709	−0.90122	−0.68870	0.29063	−0.70880	−0.93557
**SDW**	−0.81010	−0.54192	−0.50084	1.00000	0.08042	−0.68924	−0.68083	−0.61518	−0.70247	−0.47604	0.57806	0.64534	0.56314	0.58309	−0.48176	0.62897	0.25288
**RDW**	−0.32359	−0.47704	−0.41041	0.08042	1.00000	−0.35385	−0.44198	−0.33370	−0.35352	−0.54053	−0.34627	0.56983	0.52057	0.77003	−0.75475	0.41282	0.28619
**CHLO**	0.62131	0.85210	0.90000	−0.68924	−0.35385	1.00000	0.98428	0.97806	0.96181	0.69105	−0.63884	−0.91772	−0.96399	−0.80047	0.26189	−0.93248	−0.73583
**PN**	0.69822	0.80755	0.84215	−0.68083	−0.44198	0.98428	1.00000	0.94641	0.94620	0.78164	−0.52653	−0.95580	−0.97481	−0.86650	0.31791	−0.97119	−0.64459
**SC**	0.47283	0.87771	0.94077	−0.61518	−0.33370	0.97806	0.94641	1.00000	0.89458	0.69094	−0.71426	−0.83647	−0.96295	−0.72327	0.19216	−0.86540	−0.83752
**TR**	0.70295	0.81136	0.83535	−0.70247	−0.35352	0.96181	0.94620	0.89458	1.00000	0.54703	−0.52831	−0.94559	−0.88774	−0.81368	0.32811	−0.92182	−0.63441
**EL**	0.56905	0.49388	0.53686	−0.47604	−0.54053	0.69105	0.78164	0.69094	0.54703	1.00000	−0.24561	−0.71117	−0.81093	−0.76442	0.32812	−0.76274	−0.35560
**LWP**	−0.13721	−0.56849	−0.64756	0.57806	−0.34627	−0.63884	−0.52653	−0.71426	−0.52831	−0.24561	1.00000	0.30641	0.51385	0.09843	0.22563	0.41496	0.67827
**MDA**	−0.77552	−0.73156	−0.76709	0.64534	0.56983	−0.91772	−0.95580	−0.83647	−0.94559	−0.71117	0.30641	1.00000	0.91311	0.93908	−0.47216	0.94946	0.52057
**H_2O_2**	−0.54749	−0.82681	−0.90122	0.56314	0.52057	−0.96399	−0.97481	−0.96295	−0.88774	−0.81093	0.51385	0.91311	1.00000	0.85575	−0.31186	0.91498	0.75011
**PRO**	−0.74153	−0.69071	−0.68870	0.58309	0.77003	−0.80047	−0.86650	−0.72327	−0.81368	−0.76442	0.09843	0.93908	0.85575	1.00000	−0.68115	0.84505	0.43073
**SOD**	0.52001	0.36611	0.29063	−0.48176	−0.75475	0.26189	0.31791	0.19216	0.32811	0.32812	0.22563	−0.47216	−0.31186	−0.68115	1.00000	−0.24585	−0.07675
**APX**	−0.76743	−0.73134	−0.70880	0.62897	0.41282	−0.93248	−0.97119	−0.86540	−0.92182	−0.76274	0.41496	0.94946	0.91498	0.84505	−0.24585	1.00000	0.49283
**CAT**	0.03430	−0.80738	−0.93557	0.25288	0.28619	−0.73583	−0.64459	−0.83752	−0.63441	−0.35560	0.67827	0.52057	0.75011	0.43073	−0.07675	0.49283	1.00000

Plant height (PH), shoot fresh weight (SFW), shoot dry weight (SDW), root fresh weight (RFW), root dry weight (RDW), total chlorophyll (CHLO), net photosynthetic rate (PN), stomatal conductance (SC), transpiration rate (TR), electrolyte leakage (EL), leaf water potential (LWP), malondialdehyde (MDA), hydrogen peroxide (H_2O_2), proline (PRO), superoxide dismutase (SOD), ascorbate peroxidase (APX), and catalase (CAT).

Table S11. Correlation of morpho-physiological traits of eight wheat genotypes under SeNPs + heat conditions.

	PH	SFW	RFW	SDW	RDW	CHLO	PN	SC	TR	EL	LWP	MDA	H_2O_2	PRO	SOD	APX	CAT
**PH**	1.00000	0.30485	0.79293	−0.67392	−0.51700	0.84743	0.08440	0.93787	0.86392	−0.94662	−0.49112	−0.92180	−0.82216	−0.94853	−0.46490	−0.86292	−0.47287
**SFW**	0.30485	1.00000	0.53435	−0.43452	0.50753	0.02280	0.15373	0.35985	0.60079	−0.19518	−0.45598	−0.35278	−0.26445	−0.01606	−0.20764	−0.19336	−0.58270
**RFW**	0.79293	0.53435	1.00000	−0.41852	−0.33463	0.52013	0.14794	0.87278	0.81284	−0.80711	−0.58060	−0.76637	−0.55350	−0.71350	−0.04919	−0.69497	−0.50202
**SDW**	−0.67392	−0.43452	−0.41852	1.00000	0.17789	−0.44156	0.02388	−0.53002	−0.64030	0.49808	0.43255	0.46664	0.47558	0.52246	0.73818	0.29442	0.40634
**RDW**	−0.51700	0.50753	−0.33463	0.17789	1.00000	−0.38718	0.28919	−0.37376	−0.17391	0.50204	−0.17911	0.29066	0.45820	0.74467	−0.06911	0.36308	−0.24914
**CHLO**	0.84743	0.02280	0.52013	−0.44156	−0.38718	1.00000	0.30156	0.86479	0.66677	−0.90724	−0.55843	−0.91186	−0.64618	−0.83392	−0.53458	−0.90720	−0.46749
**PN**	0.08440	0.15373	0.14794	0.02388	0.28919	0.30156	1.00000	0.30172	0.33692	−0.29602	−0.68715	−0.23233	−0.02971	−0.01442	−0.23013	−0.28365	0.09209
**SC**	0.93787	0.35985	0.87278	−0.53002	−0.37376	0.86479	0.30172	1.00000	0.86069	−0.97690	−0.68655	−0.95427	−0.68953	−0.86971	−0.35701	−0.89790	−0.56708
**TR**	0.86392	0.60079	0.81284	−0.64030	−0.17391	0.66677	0.33692	0.86069	1.00000	−0.84071	−0.70145	−0.85007	−0.65945	−0.70923	−0.54559	−0.79867	−0.41534
**EL**	−0.94662	−0.19518	−0.80711	0.49808	0.50204	−0.90724	−0.29602	−0.97690	−0.84071	1.00000	0.63443	0.94578	0.70008	0.92543	0.39937	0.93204	0.42606
**LWP**	−0.49112	−0.45598	−0.58060	0.43255	−0.17911	−0.55843	−0.68715	−0.68655	−0.70145	0.63443	1.00000	0.61403	0.08412	0.32368	0.52264	0.54852	0.47369
**MDA**	−0.92180	−0.35278	−0.76637	0.46664	0.29066	−0.91186	−0.23233	−0.95427	−0.85007	0.94578	0.61403	1.00000	0.71332	0.83742	0.43873	0.96639	0.61150
**H_2O_2**	−0.82216	−0.26445	−0.55350	0.47558	0.45820	−0.64618	−0.02971	−0.68953	−0.65945	0.70008	0.08412	0.71332	1.00000	0.80481	0.23421	0.68912	0.22067
**PRO**	−0.94853	−0.01606	−0.71350	0.52246	0.74467	−0.83392	−0.01442	−0.86971	−0.70923	0.92543	0.32368	0.83742	0.80481	1.00000	0.31202	0.83102	0.27899
**SOD**	−0.46490	−0.20764	−0.04919	0.73818	−0.06911	−0.53458	−0.23013	−0.35701	−0.54559	0.39937	0.52264	0.43873	0.23421	0.31202	1.00000	0.37563	0.25617
**APX**	−0.86292	−0.19336	−0.69497	0.29442	0.36308	−0.90720	−0.28365	−0.89790	−0.79867	0.93204	0.54852	0.96639	0.68912	0.83102	0.37563	1.00000	0.43205
**CAT**	−0.47287	−0.58270	−0.50202	0.40634	−0.24914	−0.46749	0.09209	−0.56708	−0.41534	0.42606	0.47369	0.61150	0.22067	0.27899	0.25617	0.43205	1.00000

Plant height (PH), shoot fresh weight (SFW), shoot dry weight (SDW), root fresh weight (RFW), root dry weight (RDW), total chlorophyll (CHLO), net photosynthetic rate (PN), stomatal conductance (SC), transpiration rate (TR), electrolyte leakage (EL), leaf water potential (LWP), malondialdehyde (MDA), hydrogen peroxide (H_2O_2), proline (PRO), superoxide dismutase (SOD), ascorbate peroxidase (APX), and catalase (CAT).
